# Hypoxic glioblastoma-cell-derived extracellular vesicles impair cGAS-STING activity in macrophages

**DOI:** 10.1186/s12964-024-01523-y

**Published:** 2024-02-22

**Authors:** Stoyan Tankov, Marija Petrovic, Marc Lecoultre, Felipe Espinoza, Nadia El-Harane, Viviane Bes, Sylvie Chliate, Darel Martinez Bedoya, Olivier Jordan, Gerrit Borchard, Denis Migliorini, Valérie Dutoit, Paul R. Walker

**Affiliations:** 1 Translational Research Center in Onco-Hematology (CRTOH), Faculty of Medicine, University of Geneva, Geneva, Switzerland; 2 School of Pharmaceutical Sciences, University of Geneva, Geneva, Switzerland; 3 Swiss Cancer Center Léman, Geneva and Lausanne, Switzerland; 4 Agora Cancer Research Center, Lausanne, Switzerland

**Keywords:** cGAS-STING pathway, Extracellular vesicles, Glioblastoma, Hypoxia/ miRNA

## Abstract

**Background:**

Solid tumors such as glioblastoma (GBM) exhibit hypoxic zones that are associated with poor prognosis and immunosuppression through multiple cell intrinsic mechanisms. However, release of extracellular vesicles (EVs) has the potential to transmit molecular cargos between cells. If hypoxic cancer cells use EVs to suppress functions of macrophages under adequate oxygenation, this could be an important underlying mechanism contributing to the immunosuppressive and immunologically cold tumor microenvironment of tumors such as GBM.

**Methods:**

EVs were isolated by differential ultracentrifugation from GBM cell culture supernatant. EVs were thoroughly characterized by transmission and cryo-electron microscopy, nanoparticle tracking analysis (NTA), and EV marker expression by Western blot and fluorescent NTA. EV uptake by macrophage cells was observed using confocal microscopy. The transfer of miR-25/93 as an EV cargo to macrophages was confirmed by miRNA real-time qPCR. The impact of miR-25/93 on the polarization of recipient macrophages was shown by transcriptional analysis, cytokine secretion and functional assays using co-cultured T cells.

**Results:**

We show that indirect effects of hypoxia can have immunosuppressive consequences through an EV and microRNA dependent mechanism active in both murine and human tumor and immune cells. Hypoxia enhanced EV release from GBM cells and upregulated expression of miR-25/93 both in cells and in EV cargos. Hypoxic GBM-derived EVs were taken up by macrophages and the miR-25/93 cargo was transferred, leading to impaired cGAS-STING pathway activation revealed by reduced type I IFN expression and secretion by macrophages. The EV-treated macrophages downregulated expression of M1 polarization-associated genes *Cxcl9*, *Cxcl10* and *Il12b*, and had reduced capacity to attract activated T cells and to reactivate them to release IFN-γ, key components of an efficacious anti-tumor immune response.

**Conclusions:**

Our findings suggest a mechanism by which immunosuppressive consequences of hypoxia mediated via miRNA-25/93 can be exported from hypoxic GBM cells to normoxic macrophages via EVs, thereby contributing to more widespread T-cell mediated immunosuppression in the tumor microenvironment.

**Supplementary Information:**

The online version contains supplementary material available at 10.1186/s12964-024-01523-y.

## Background

Glioblastoma (GBM) is the most lethal primary brain tumor and one of the most aggressive types of solid cancer [1]. In GBM, there is a notable level of heterogeneity within and between tumors. The diverse components of the GBM tumor microenvironment (TME) interact with each other, intensifying this heterogeneity, for example, through interactions of stromal cells, endothelial cells, as well as many different innate and adaptive immune cells (such as tumor-associated macrophages and microglia, neutrophils, natural killer cells, and T and B lymphocytes). Together, these cells work to provide the tumor with numerous cues that encourage progression and invasion rather than significant immune control. Additionally, the presence of hypoxia and its consequences such as low pH, further alters interactions between the various cell populations, worsening prognosis [2] and supporting immune evasion through diminished cytotoxic T cell infiltration, proliferation, or activity [3].

In addition to soluble factors and cell-to-cell contacts that have been explored for decades, intercellular communication within the GBM TME can also be mediated by extracellular vehicles (EVs). EVs can carry a variety of cargos, including RNAs, proteins, lipids and DNA, which can be taken up by other cells, both in the direct vicinity of the source cell and at distant sites in the body via biofluids, and elicit a variety of functionally important responses [4–6]. GBM EVs can regulate the expression of various genes in the recipient cells by surface molecules interactions or through their cargo in the TME and even to more distal cells through cerebral spinal fluid (CSF) and blood [7]. EV uptake occurs through several mechanisms including plasma membrane fusion, endocytosis, micropinocytosis, phagocytosis, as well as direct internalization mediated by lipid rafts [8]. GBM EVs can interfere with signaling pathways through the delivery of coding and non-coding RNA cargos [9], with miRNAs, being particularly well studied [10]. GBM cells can affect both tumor and stromal cells in the TME, and efficiently recruit infiltrating monocytes from the blood, [11] and brain resident myeloid cells, microglia, from other areas of the brain [12]. Subsequently, as suggested in several studies, GBM-delivered EVs and their miRNA cargo can be involved in the repolarization of myeloid cells in vitro [13, 14] and can even directly reprogram microglial cells in the TME [6]. Regarding T and NK cells (major anti-tumor effector cells), GBM-derived EVs were reported to carry surface molecules (including PD-L1) that can reduce anti-tumor effector functions of these lymphocytes [15, 16]. GBM-EVs also have the potential to impact non-immune cells in the TME. For astrocytes, they were reported to reprogram their metabolism and drive them towards tumor-promoting functions [17, 18]. Vascularization can also be impacted by GBM-derived EVs, through post-transcriptional modification in endothelial cells by miR-9 transfer [19]. GBM-EVs are also involved in radiation-resistance through cargos of specific miRNAs targeting the PTEN pathway [20]. Indeed, many of the cancer-associated miRNAs (oncomiRs) are released by GBM cells as EV cargos, which adds additional layers of complexity and underscores the possibility of miRNAs acting in a paracrine manner. One of the most studied GBM-associated oncomiRs is miR-21, inducing cancer cell proliferation and tumor growth [21] Moreover, miR-21 can also be exported as an EV cargo and promote an immune-suppressive phenotype in recipient myeloid cells [22]. miR-25 and miR-93a are well-studied oncomiRs involved in the development of many tumor types including GBM [23–25]. GBM cancer cell intrinsic miR-25 upregulation promotes cell proliferation, and it is overexpressed in more than 90% of human GBM biopsies compared to normal brain tissues [26]. Among the many potential target genes of miR-25/93, in breast cancer cells, *CGAS* was shown to be indirectly downregulated through *NCOA3* [27].

The cGAS-STING pathway is the principle cellular cytosolic double-stranded DNA (dsDNA) sensor, leading to type I IFN release and facilitating innate immune responses to infections and cancer [28], and is required for efficacy of immune checkpoint blockade immunotherapy in cancer models [29]. Type I IFN release facilitates antigen presentation and T cell effector functions, leading to enhanced killing of tumor cells [30, 31]. The endogenous stimulation of cGAS-STING pathway in cancer can occur through uptake of tumor cell derived DNA, which will be abundant in hypoxic and necrotic zones, conditions that promote DNA damage and nuclear leaks [32]. The therapeutic potential of robust cGAS-STING pathway activation in GBM has been demonstrated by use of synthetic cGAMP as a STING agonist that promotes anti-tumor immunity, implicating both T cells and innate immune cells such as macrophages [33].

GBM is highly infiltrated by myeloid cells that include microglia and macrophages with these infiltrating myeloid cells constituting up to 50% of the tumor mass [34, 35]. These putatively immunosuppressive cells can limit efficacy of immune checkpoint inhibitors to rejuvenate functionality of tumor infiltrating T cells [36]. Tumor infiltrating macrophages originate from the differentiation of peripheral monocytes recruited and polarized to an M1-like or M2-like state in response to a variety of tumor-derived cytokines, chemokines and DNA as well as other non-soluble factors such as viruses and bacteria that can be present in the TME [37–39]. Macrophages designated as M1-like are classically activated, typically by IFN-γ or lipopolysaccharide (LPS) and produce pro-inflammatory cytokines. In contrast, M2-like macrophages are alternatively activated by exposure to certain cytokines (IL-4 and IL-13) and are associated with wound healing and tissue repair [40]. In the context of cancer, M1-like macrophages are considered anti-tumoral while M2-like macrophages exhibit pro-tumoral properties [41]. However, with better understanding of the population, it is highly likely that tumor-infiltrating macrophages in GBM are composed of heterogeneous subpopulations [42]. Notably, the cGAS-STING pathway is implicated in macrophage polarization [43–45], in addition to the EV-mediated polarization modulation as already discussed. Some cancer cells were described to bypass the cGAS-STING-dependent immune response by either modulating cGAS expression, or by suppressing its functions, thereby participating in immune evasion [27, 46]. Nevertheless, innate immune cells are major type I IFN producers that influence cancer immunosurveillance [47, 48], therefore it is important to understand the cGAS-STING pathway mechanisms in these cells and whether it can be modulated indirectly by factors present in the TME. Indeed, there have been no breakthroughs in GBM treatment using therapies targeting the GBM cells, suggesting that new therapies should consider not only the cancer cells, but also other cells or features of the TME in order to achieve clinical impact.

Here, to better understand how small zones of hypoxia can so negatively affect surrounding cells in less hypoxic areas of tumor, we elucidated a hypoxia-dependent mechanism that can amplify immunosuppression. We show that the elevated production of miR-25 and miR-93 within hypoxic GBM cells leads to EV-shuttled transfer of these miRNAs to normoxic macrophages; this resulted in suppression of cGAS expression, and a deficient response to tumor-cell derived DNA. Type I IFN release and expression of M1 associated genes was impaired, as was chemoattraction of T cells, which correlated with reduced CXCL9/10 expression.

## Methods

### GBM cell cultures

Human Ge738, Ge982, Ge975, Ge904 and Ge835 cell lines were used at low passage (<P7) and were originally generated from resection of IDH wild type (WT) GBM. All patients gave written informed consent for sample collection. The study was conducted in accordance with the Declaration of Helsinki, and the protocol was approved by the Ethics Committees of Geneva University Hospitals and the Canton of Geneva (CCER) (03–126). The human LN18 line was obtained from American Type Culture Collection (ATCC); mouse SB28 and GL261 cell lines were kindly provided by H. Okada, University of California, San Francisco (UCSF), USA. All cell lines were cultured in serum-containing Dulbecco’s Modified Eagle Medium (DMEM) media (Gibco) supplemented with 10% Fetal Bovine Serum (FBS) that was centrifuged for 12 h at 100,000 g to deplete EVs. GBM cell lines were exposed to atmospheric O_2_ conditions in a conventional hood and incubator, or to 1% O_2_ using a Ruskinn 300 InVivO2 hypoxia workstation (Baker) for 24–48 h. Media were pre-equilibrated to the desired oxygen level by flushing with the corresponding gas mix. All cell lines tested negative for mycoplasma.

### Human macrophage generation and differentiation and T cell isolation

Cells were collected from buffy coats of healthy donors provided by the Transfusion center of the Geneva University Hospital. On Day 0 blood was diluted 1:1 with PBS and 15 ml were delicately poured on 15 ml Lymphoprep (STEMCELL Technologies) in a 50 ml tube and centrifuged at 433×g for 30 minutes without brake. The top layer (plasma) was removed and the peripheral blood myeloid cells from the intermediate layer were collected and washed twice with PBS by centrifugation (1200 rpm for 10 minutes). Next, the cells were passed through a 70 μM filter, counted and resuspended in EDTA/BSA/PBS buffer according to manufacturer’s instructions for CD14 microbead positive selection. Monocytes were then selected using human CD14 Microbeads and MACS LS columns (according to CD14 MicroBeads human protocol from Miltenyi Biotec). Cells were then plated in bacteriological grade petri dishes (GREINER; sterilized by UV for 30 minutes) at a concentration of 3 × 10^6^ cells per dish. The culture medium (RPMI-1640) was supplemented with 10% FBS, Penicillin (100 U/ml), Streptomycin (100 μg/ml), HEPES (10 mM), 1x Non-essential amino acid mix (ThermoFisher), 1x Sodium Pyruvate (Gibco) β-mercaptoethanol (50 μM) and human recombinant M-CSF (Peprotech) at a concentration of 10 ng/ml. On day 2 and 5, the cells were washed once with PBS and fresh culture medium supplemented with M-CSF was added. T cells were collected from buffy coats of healthy donors provided by the Transfusion center of the Geneva University Hospital. Buffy coats were diluted 1:1 with PBS and 15 ml were delicately poured on 15 ml Lymphoprep (STEMCELL Technologies) in a 50 ml tube and centrifuged at 433×g for 30 minutes without brake. The top layer (plasma) was removed and the peripheral blood mononuclear cells from the intermediate layer were collected and washed twice with PBS by centrifugation (1200 rpm for 10 minutes). Next, the cells were passed through a 70 μM filter, counted and resuspended in EDTA/BSA/PBS buffer according to manufacturer’s instructions for CD3 microbead positive selection using human CD3 Microbeads and MACS LS columns (according to CD3 MicroBeads human protocol from Miltenyi Biotec). The CD3^+^ T cells were then washed and frozen in FBS/10% DMSO freezing media until used.

### Mouse macrophages

Bone marrow derived macrophages (BMDM) were isolated either from non-manipulated wild type C57BL/6 mice or after in vivo activation. For non-manipulated mice, mice were sacrificed, and their bone marrow isolated by flushing each femoral bone immediately following the bone cut. The cell suspension was centrifuged 5 min at 350 g then resuspended and passed through a cell strainer (70 μm). Cells were then counted and cultured in RPMI-1640 media supplemented with 10% FBS, Penicillin (100 U/ml), Streptomycin (100 μg/ml), HEPES (10 mM), 1x Non-essential amino acid mix (ThermoFisher), 1x Sodium Pyruvate (Gibco) β-mercaptoethanol (50 μM) and mouse recombinant M-CSF (Peprotech) at a concentration of 10 ng/ml.

Macrophages were differentiated in vitro in supplemented RPMI 1640 medium as follows: for M0, M1 and M2 macrophages, M-CSF was added at day 0, 3 and 5 at final concentration of 10 ng/ml; for M1 macrophages IFN-γ (20 ng/ml) and LPS (100 ng/ml) (Immunotools) were added at day 5; for M2 macrophages IL-4 (20 ng/ml) and IL-13 (20 ng/ml) (Immunotools) were added at day 5. After a further 2 days (day 7), macrophages were harvested, washed, and used as indicated.

For in vivo activated macrophages, we used elicited peritoneal macrophages [49]. An inflammatory reaction was induced in the peritoneum of mice by injection of sterile Brewer thioglycollate medium (MedChemExpress); after 4 days, mice were euthanized and peritoneal macrophages were harvested for in vitro testing. All animal experimental studies were reviewed and approved by institutional — Direction de l’expérimentation animale, Geneva, Switzerland — and cantonal — Direction générale de la santé, Geneva, Switzerland — veterinary authorities in accordance with Swiss Federal law under the authorization 32229/ GE-33-20.

### Generation of miR-25/93 knockout SB28 cells

For knockout (KO) of miR-25 in murine SB28, CRISPR/Cas9-based gene KO was performed using eSpCas9 (Sigma-Aldrich) according to the manufacturer’s protocol. The following pairs of synthetic gRNAs were used: set1 *miR-25 5′-CGGAGACUUGGGCAAUUGC-3′* set2 *mir-93 5′-UAGCACUUCCCGAGCCCCC-3′* and set3 *Negative control 5’CGCGAUAGCGCGAAUAUAUAUU-3′*. Briefly, SB28 cells were cultured at 50–60% confluency in 6 well plates 24 h in advance. The transfection of gRNA was achieved by mixing SygRNA crRNA (SigmaAldrich) and tracrRNA on ice with CAS9 protein (SigmaAldrich). The mix was incubated for 30 minutes for Cas9 RNP complex formation. The incubation was followed by addition of TransIT-CRISPR transfection agent (SigmaAldrich) mixed gently and incubated at RT for 20 minutes. The final complex was distributed to cells and incubated 24 h before the media was replaced and cells were cultured for an additional 24 h. Finally, the cells were collected, and single clones were expanded in 96-well plates, then tested for miR-25/93 expression using RT-qPCR. The clones that did not express miR-25/93 were selected and further used.

### DNA challenge and miR-25 transfection assay

BMDMs were isolated polarized and cultured as described above. 5 × 10^4^ BMDMs (M0, M1 or M2) per well were seeded in 96 well plates. Total DNA isolated from SB28 cells was mixed with Lipofectamine 3000 (ThermoFisher) and transfected (5 μg/ml) into BMDMs according to manufacturer’s protocol. The cells were then incubated for 24 h under normoxic (21%) or hypoxic (1%) O_2_ conditions. Similarly, the miR-25 mimic and miR-scramble (ThermoFisher) controls were transfected to elicited peritoneal macrophages using Lipofectamine 3000 (ThermoFisher) in 24 well plates. Briefly, 5 × 10^5^ cells were seeded for 24 h in RPMI medium supplemented with 10% FBS, penicillin (100 U/ml), streptomycin (100 μg/ml), HEPES (10 mM), 1x non-essential amino acid mix (ThermoFisher), 1x sodium pyruvate (Gibco) β-mercaptoethanol (50 μM). After 24 h Lipofectamine® 3000 reagent and Opti-MEM Medium (ThermoFisher) were mixed and combined to the mixture in Opti-MEM® Medium, P3000 Reagent (ThermoFisher) and miRNA-25 mimic or miR-scramble and incubated for 5 minutes. The final mix was added dropwise to the wells containing cells and incubated for an additional 24 h prior to RT-qPCR.

### CD4^+^ T cell activation in vitro

Non-manipulated OT-II mice were sacrificed and splenocytes collected and cultured in DMEM media (Gibco) supplemented with 6% glucose, 10% FBS, HEPES (10 mM), β-mercaptoethanol (50 μM), amino acid mix (1.4 mg/mL Arg, 3.2 mg/mL Asp). Cells were expanded for 7 days with CD3/CD28 coated beads (ThermoFischer) in 1:1 bead/cell ratio and the addition of IL-2 (50 U/ml) (Immunotools) on day 3 and 5.

### RNA extraction and qPCR

0.5 μg of total RNA isolated with Total RNA Mini kit (A&A Biotechnology, Poland) was used to synthesize cDNA with a mix of random hexamers – oligo d(T) primers and PrimerScript reverse transcriptase enzyme kit (Takara bio inc.) following supplier’s instructions. SYBR green assays were designed using the program Primer Express v 2.0 (Applied Biosystems) with default parameters. Amplicon sequences were aligned against the mouse/human genome by BLAST to ensure that they were specific for the gene being tested. Oligonucleotides were obtained from Invitrogen/Thermo Fisher (Supp Table 1). The efficiency of each design was tested with serial dilutions of cDNA. PCR reactions (10 μl volume) contained diluted cDNA, 2 x Power SYBR Green Master Mix (Applied Biosystems, USA) and 300 nM of forward and reverse primers. PCR was performed on an SDS 7900 HT instrument (Applied Biosystems) with the following parameters: 50 °C for 2 minutes, 95 °C for 10 minutes, and 45 cycles of 95 °C 15 seconds-60 °C 1 minute. Each reaction was performed in three replicates on a 384-well plate. Raw Ct values obtained with SDS 2.2 (Applied Biosystems) were imported in Excel and normalization factor and fold changes were calculated using the GeNorm method [50].

Total microRNA was obtained from cells and corresponding EVs (after isolation and before resuspension in PBS, see EV isolation protocol) using a microRNA isolation kit (A&A Biotechnology, Poland) according to the manufacturer’s protocol. cDNA templates were prepared using TaqMan Advanced miRNA cDNA synthesis kit (ThermoFischer Scientific, USA) according to the manufacturer’s protocol.

### EV isolation and NTA/fNTA characterization

EVs were isolated from supernatants of mouse GBM cell lines SB28 and GL261, and human derived GBM cell lines LN18, Ge738, Ge982, Ge975, Ge904 and Ge835. The cells (2.5 × 10^5^) were cultured in 15 ml of medium in T75 flasks (TPP) in triplicates for 24 h and the media was collected for EV isolation The isolation procedure was based on a previously described protocol [51] with modifications. Briefly, culture medium was centrifuged at 300 g for 10 min to pellet the cells and large cell debris. The supernatant was then centrifuged for 10 min at 2000 g to remove dead cells and small cell debris. Finally, EVs were pelleted by ultracentrifugation at 100,000 g for 70 min and washed with PBS once, then pelleted again by ultracentrifugation at 100,000 g for another 70 min. The EV containing pellet was resuspended in PBS for subsequent tests. Size and concentration of EVs were quantified using Nanoparticle Tracking Analysis (NTA) (Particle Metrix, Germany). For further functional experiments, EV:recipient cell ratios were normalized to 3000 nanoparticles (measured by NTA) per recipient cell.

### Electron microscopy and Cryo-EM

EV samples were placed on glow discharged 200 mesh copper grids coated with formvar and carbon. After 1 min absorption, samples were dried, washed three times and stained with aqueous 2% uranyl acetate for 1 min. The stain was blotted dry from the grids with filter paper and samples were allowed to dry. Samples were then examined in a Tecnai 20 transmission electron microscope (FEI Company, Netherlands) at an accelerating voltage of 80 kV. Digital images were obtained using the AMT Imaging System (Advanced Microscopy Techniques Corp., USA).

Cryo-EM was used for direct visualization of extracellular vesicles. To prepare samples for cryo-EM study, lacey carbon EM grids were glow-discharged (30 s, 25 mA) in a Pelco EasiGlow system. An aliquot (3 μL) of the EV suspension in PBS was applied to the carbon side of an EM grid, which was then blotted for 3.0 s and plunge-frozen into the precooled liquid ethane. This procedure results in embedding the samples in a thin layer of amorphous ice to preserve them in their native state and to protect them from radiation damage. The samples were studied in a Talos Arctica (200 KeV, FEG) cryo-electron microscope.

### SDS-PAGE and Western blotting

SDS-PAGE was performed as follows. For cell lysates, protein concentrations were measured at 562 nm using the bicinchoninic acid assay kit (Pierce) and 20 μg of protein was mixed with 4× NuPAGE LDS sample buffer. For EVs extracts, proteins were also mixed with 4× NuPAGE LDS sample buffer. For reducing conditions, samples were supplemented with 10× NuPAGE reducing agent. All samples were then denatured at 95 °C for 10 min before being added to a 12% polyacrylamide Bis-Tris gel (Life Technologies) and electrophoresed at 200 v for 70 min in MOPS SDS Running buffer (LifeTechnologies). Following electrophoresis, the gel was transferred onto polyvinylidene fluoride (PVDF) membrane using the Semi-Dry Blotting system (LifeTechnologies™) according to manufacturer’s instructions. PVDF membranes were probed with primary antibodies specific for Hsp70 [EPR16892] (Abcam), TSG101 [EPR7130(B)] (Abcam) or Calnexin [EPR3633(2)] - ER Membrane Marker (Abcam) and Goat anti-rabbit secondary antibody conjugated with HRP (LifeTechnologie, 1:1000). The membrane was then incubated with Amersham ECL Prime Western Blotting Detection Reagent for 5 minutes at room temperature and protein were visualized using Fusion FX (Vilber Lourmat).

### Fluorescence microscopy

Previously isolated and cultured macrophages (BMDMs, thioglycolate-elicited peritoneal macrophages (EPMs) or human macrophages) were collected washed and cultured for 6 hours in a chamber slide system (ThermoFisher). EVs were membrane stained with BODIPY (ThermoFisher) TR ceramide, final dye concentration was 10 μM and EVs were incubated at 37 °C for 20 min. The excess unincorporated dye from the labeled EVs was removed by two washes, centrifuging at 120′000 x g for 1 h.

Labeled EVs were mixed with macrophages and cultured on chamber slides for 6 (at 37 °C or 4 °C), 12, or 24 h. After that the media was removed and the slides with the attached macrophages were washed with PBS, fixed with PFA and stained with DAPI, F4/80, CD11b or CD68. The imaging was performed using LSM700 confocal microscopy and the images were analyzed with ZEN Imaging Software (Zeiss, Germany).

## ELISA

6 × 10^4^ cells were cultured for 24 h, supernatants were removed and assessed for interferon β (IFN-β) content. 96 well plates were coated with anti-IFN β (clone RMMB-1, PBL) (50ul/well) and incubated at 4 °C overnight. The next day, standard dilutions (1000 U/ml − 7.8 U/ml) of mouse IFN β (PBL) and samples were added (50 μl/well). Two hours later, the detection antibody (PBL) was added, followed by secondary HRP-linked anti-rabbit IgG (Cell Signaling). The reaction was revealed by addition of the TMB substrate (BD OptEIA) and stopped by addition of 25 μl of 2 N H_2_SO_4_. Optical absorbance was measured at 450 and 570 nm using a microplate reader (Biotek SynergyMx) and IFN-β levels expressed as U/ml. Results are presented as mean ± SD of triplicate experiments (*n* = 5).

IFN-γ production was measured from supernatants of co-cultures of M0 or M1 polarized BMDMs and OT-II CD4^+^ T cells. Briefly, BMDMs were collected and washed from the dishes in which they were cultured. The cells were counted, and half of the cells were pulsed with 10 μM OVA peptide (ISQAVHAAHAEINEAGR) (Proteogenix) for 1 h at 37 °C and the other half were incubated without added peptide. Then, 5 × 10^4^ BMDMs per well (OVA pulsed or unpulsed control) were plated in a 96 well plate and 10^5^ of OT-II CD4^+^ T cells per well were added in each well for 24 h at 37 °C, 5% CO_2_ and atmospheric oxygen (21% O_2_). After 24 h, the supernatant from the co-cultures was collected and used for IFN-γ measurement using a mouse IFN-γ ELISA Set (BD Bioscience) according to manufacturer’s protocols.

### Cytokine multiplex assay

CXCL10 was measured using the LEGENDplex (Biolegend) Mouse Anti-Virus Response Panel bead-based multiplex assay using manufacturer’s instructions. Briefly, 5 × 10^4^ BMDMs (previously polarized to M1 or unpolarized M0 with or without the addition of hypoxic GBM EVs) were seeded on the bottom of 24 well plate for 2-3 h until attached. CD4^+^ T cells were isolated from OT-II mouse spleens, activated and expanded as described above. 10^5^ CD4^+^ T cells were collected and cocultured with BMDMs for 12 h. The media from each well was collected, centrifuged at 1200 rpm for 10 minutes, and used for CXCL10 detection.

### Migration assay

For the mouse T cell migration assay, CD4^+^ T cells were isolated from OT-II mouse spleens, activated and expanded as described above. After 7 days CD4^+^ T cells were collected and used. CD4^+^ T cells pretreated with pertussis toxin (100 ng/ml) were used as a negative migration control; for a positive control mouse CXCL10 (30 ng/ml) was added directly to the well. 5 × 10^4^ M0 and M1 generated as described above from BMDMs (EVs treated, or vehicle treated) were seeded in 24 well plates for 3 h. Transwell cell culture inserts (Greiner Bio-One) were placed in each well and 10^5^ OT-II CD4^+^ T cells were added in the inserts. After 6 h the inserts were removed, the CD4^+^ T cells that migrated from inserts were counted. For the human T cell migration assay CD3^+^ T cells were isolated from buffy coats as described above. CD3^+^ T cells pretreated with pertussis toxin (100 ng/ml) were used as a negative migration control; for a positive control, human CXCL10 (10 ng/ml) was added directly to the well. 5 × 10^4^ macrophages generated as described above from MDMs (EV treated, or vehicle treated) were seeded in 24 well plates for 3 h. Transwell cell culture inserts (Greiner Bio-One) were placed in each well and 10^5^ CD3^+^ T cells were added in the inserts. After 8 h the inserts were removed, the CD3^+^ T cells that migrated from inserts were counted. Migration is expressed as a migration index: (number of cells on the undersurface of the membrane divided by the total number of cells on both surfaces of the membrane) × 100.

### Statistics

All results are expressed as mean ± SD. Statistical analyses were performed with Prism version 9.0 (GraphPad Software). For analysis of data between two groups, an unpaired t-test (2-tailed unless otherwise stated) was used. The *p* values < 0.05 were considered significant. All experiments were reproduced at least twice.

## Results

### GBM cells upregulate EV production under hypoxia

**Fig. 1:**
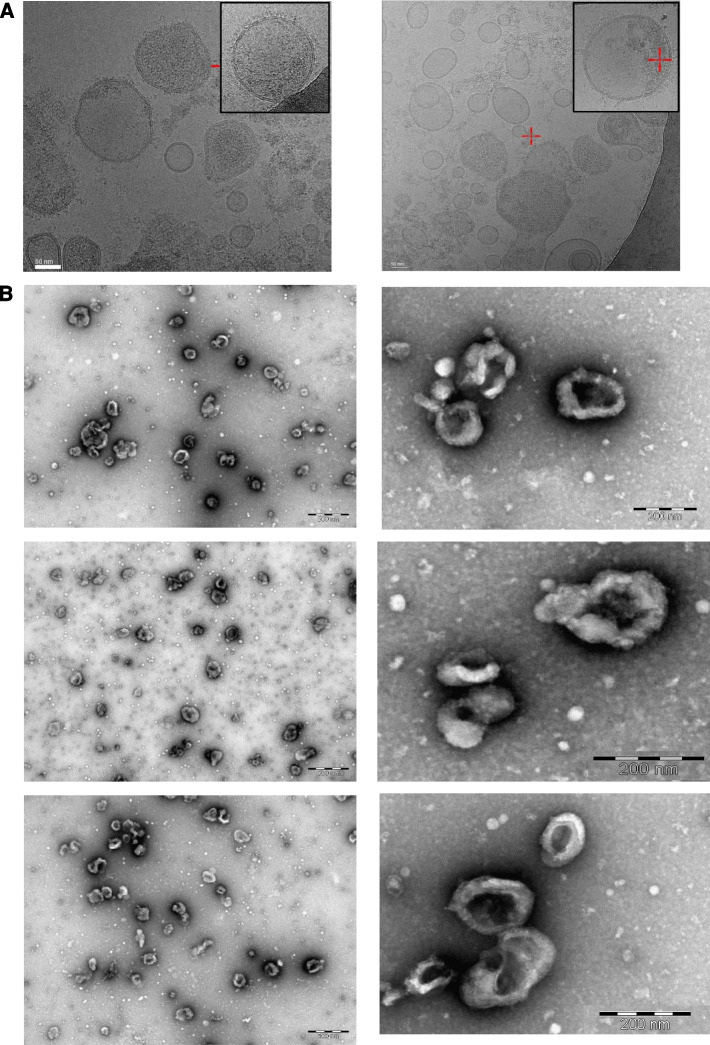
Electron microscopy characterization of GBM-derived EVs from normoxic or hypoxic culture. **A** Electron cryo-microscopy (cryo-EM) imaging of EVs secreted by murine (SB28, left) and human (Ge904, right) GBM cell lines. Pictures are representative of at least 3 images. **B** Transmission Electron Microscopy (TEM) imaging of EVs secreted by murine (SB28, top row) and human (Ge904, middle row and Ge835 lower row) GBM cell lines. Pictures are representative of at least 6 images

**Fig. 2:**
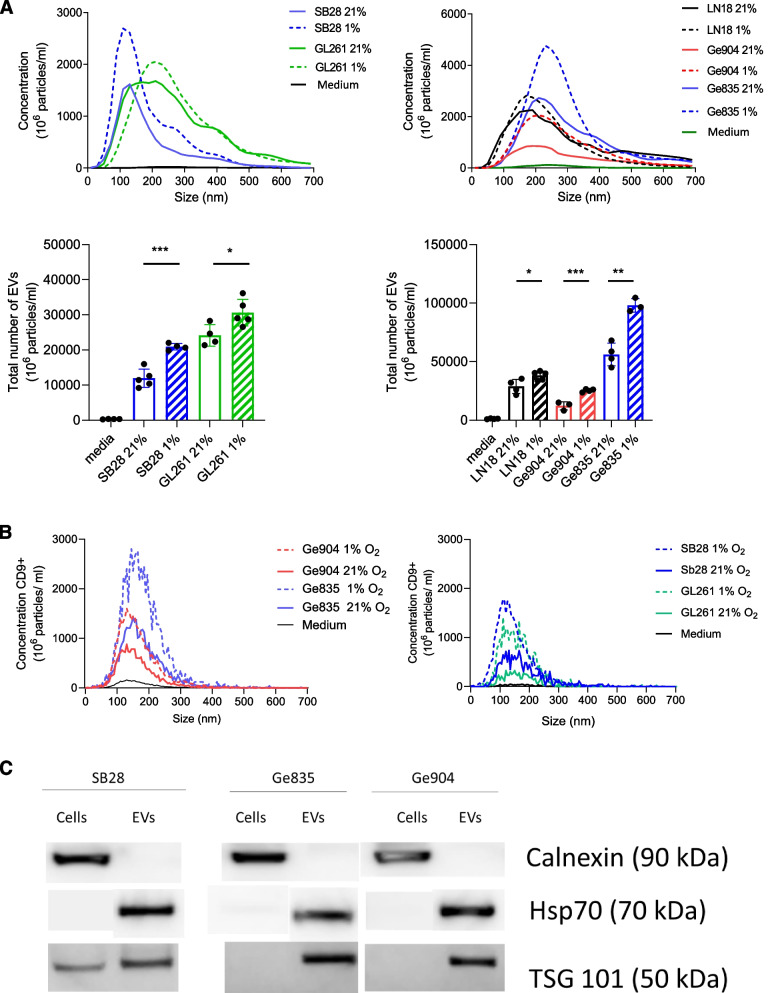
NTA characterization of GBM-derived EVs from normoxic or hypoxic culture. **A** NTA profiles of EVs isolated from murine SB28 and GL261 cell lines (left) and from human Ge904, Ge835 and LN18 GBM cell lines (right) cultured in hypoxic (1% O_2_) or normoxic (21% O_2_) conditions. (upper panels). Total number of EVs measured by NTA from murine (SB28 and GL261) (left) and from human (Ge904, Ge835 and LN18) GBM cell lines (right) cultured in hypoxic (1% O_2_) or normoxic (21% O_2_) conditions (lower panel). EV depleted culture media was used as control. The calculated size distribution in the upper panels is depicted as a mean from three experiments and three measurements. In the lower panels, data is presented as the mean ± SD of three independent experiments and comparisons were made using an unpaired t test. *p < 0.05, **p < 0.005, ***P < 0.001. **B** fNTA profiles of EVs isolated from murine SB28 and GL261 cell lines (left) and from human Ge904, Ge835 and GBM cell lines (right) cultured in hypoxic (1% O_2_) or normoxic (21% O_2_) conditions. The EVs were stained with APC conjugated anti-CD9 antibody (anti-mouse or anti-human correspondingly). EV depleted culture media was used as control. The calculated size distribution is depicted as a mean from three experiments and three measurements. **C** Western blot analysis of cells and EVs from murine (SB28) and human (Ge904 and Ge835) cell lines

GBM EVs can carry various cargos with immunosuppressive functions, thus we analyzed the EV content of mouse and human GBM cell lines in vitro. EVs were isolated from the supernatant of mouse SB28 and GL261 and human LN18, Ge904 and Ge835 GBM cell lines cultured in hypoxic (1% O_2_) or normoxic (21% O_2_) conditions. GBM-derived EVs were subjected to cryo-EM imaging, revealing a spherical nanovesicular morphology without noticeable damage (Fig. 1A). Transmission electron microscopy (TEM) with negative staining showed typical EV features [52], i.e. a characteristic doughnut-like shape and size (Fig. 1B and Supplementary Fig. 1) that was reported in other studies [53]. The combination of high-speed and ultracentrifugation allows us to collect all the EVs types (exosomes, microvesicles and apoptotic bodies) secreted from the GBM cells. This method of EV collection is important when studying the functional role of the EVs in the TME where all the types of EVs are present and can potentially affect the cells in the TME. Next, to accurately quantify EVs in these samples, we used Nanoparticle Tracking Analysis (NTA), a highly sensitive method that calculates the size and the concentration of the particles based on their Brownian motion [54]. In normoxic conditions, differences in median EV size were observed between the mouse GBM lines, as well as, to a lesser extent, in human GBM lines (Fig. 2A, upper panels and Supplementary Fig. 4A). Peak size was not affected by culture in hypoxic conditions and includes vesicles with an exosomal size profile (30–150 nm), as well as larger microvesicles. On the contrary, when GBM cells were exposed to hypoxic conditions, the number of EVs secreted by the GBM cells also significantly increased. This hypoxia enhanced EV secretion was observed in all of the cell lines tested and was particularly high in SB28 and Ge904 cell lines (Fig. 2A, lower panels).

The EV profile was further investigated using fluorescent Nanoparticle Tracking Analysis (fNTA) and CD9 immunolabeled EVs (Fig. 2B). Particle size distributions varied between EV sources (different cell lines) but closely resembled the non-fluorescent NTA profiles. The concentration of fluorescent (CD9^+^) particles represented around 30% of the total number of particles measured by non-fluorescent NTA analysis. This indicates that out of 3000 particles that we used in the invitro assays minimum 1000 (estimated to previously to be produced by single cancer cell for 24 h [55]) are of EV nature. Furthermore, Western blot (WB) analysis of EVs indicated enrichment of EV-associated proteins, such as TSG-101 and Hsp70 and lack of Calnexin (ER contamination), in EV preparations compared to GBM cells (Fig. 2C). Overall, we determined that human and mouse GBM cell lines i) secrete EVs with a broad size profile, including exosomes, ii) secrete elevated levels of these EVs under hypoxia and iii) secrete EVs that are positive for tetraspanins (CD9) and other EV-associated protein markers.

### Macrophages internalize hypoxic GBM-derived EVs

**Fig. 3:**
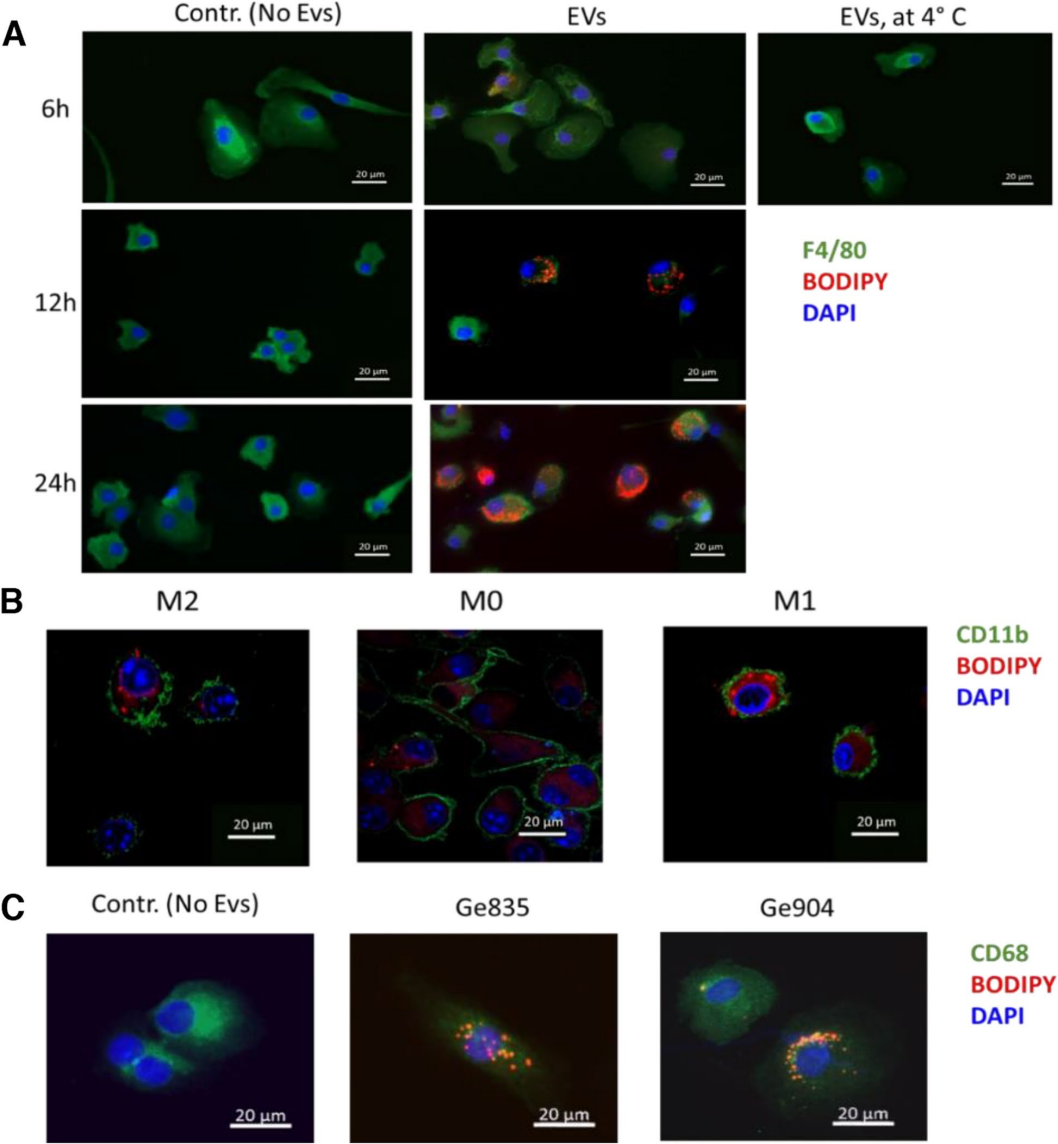
GBM-derived EVs are internalized by macrophages. **A** Kinetics of macrophage uptake of BODIPY membrane-labelled (red) EVs derived from hypoxic SB28 cells at the indicated times. EPMs were stained for F4/80 (green) and DAPI (blue). Negative control: macrophages incubated with addition of non-labelled EVs. For passive membrane uptake control, macrophages were incubated at 4 °C for 6 h with BODIPY membrane-labelled EVs. **B** Uptake of hypoxic SB28-derived EVs labeled with BODIPY (red) by BMDMs, M0, or after polarization to M1 and M2. BMDMs were incubated with labeled EVs for 24 h at 37 °C and stained for CD11b (green) and DAPI (blue). **C** Uptake of BODIPY-labeled (red) EVs from hypoxic Ge835 or Ge904 cells by human monocyte-derived macrophages (MDMs) stained for CD68 (green) and DAPI (blue). Control: MDMs without EVs. MDMs were incubated with the corresponding labeled EVs for 24 h at 37 °C before fixation and imaging. Pictures are representative of a minimum of 3 images per group

To explore whether hypoxia-induced EVs released by GBM cells interact with macrophages only at the cell surface, or also intracellularly, we used confocal microscopy to image EV internalization by EPMs. Membrane-labelled EVs were internalized by EPMs starting 6 h after of co-culture with EVs derived from hypoxic SB28 cells (Fig. 3A). Increasing incubation time (12 and 24 hours) resulted in higher EV uptake (Fig. 3A). Uptake was virtually absent in macrophages incubated at 4 °C for 6 h, suggesting an energy-dependent uptake process rather than passive membrane passage as previously reported [56, 57]. Altogether, our results are consistent with an endocytic process rather than membrane fusion as EV uptake was time and temperature dependent.

We next evaluated the capacity of resting M0 and M1- or M2- in vitro polarized mouse bone marrow derived macrophages (BMDMs) to uptake hypoxic SB28 GBM- derived EVs. After 24 h of co-culture, all macrophages internalized labeled EVs (clearly recognizable BODIPY stained vesicle-like structures) regardless of their polarization status (Fig. 3B). In contrast, direct staining of macrophages with BODIPY for 12 h gave diffuse staining (Supplementary Fig. 2). To extend our findings to human cells, we used EVs from hypoxic human GBM lines Ge835 and Ge904, which we incubated for 24 h with human monocyte derived macrophages (MDMs). Confocal images of the MDMs stained for CD68 and DAPI revealed that EVs derived from both Ge835 and Ge904 were internalized by MDMs (Fig. 3C). Overall, we observed that mouse and human macrophages have the ability to internalize hypoxic GBM-derived EVs, a process that occurs regardless of the macrophage polarization status.

### GBM cells package miR-25/93 in EVs under hypoxia

**Fig. 4:**
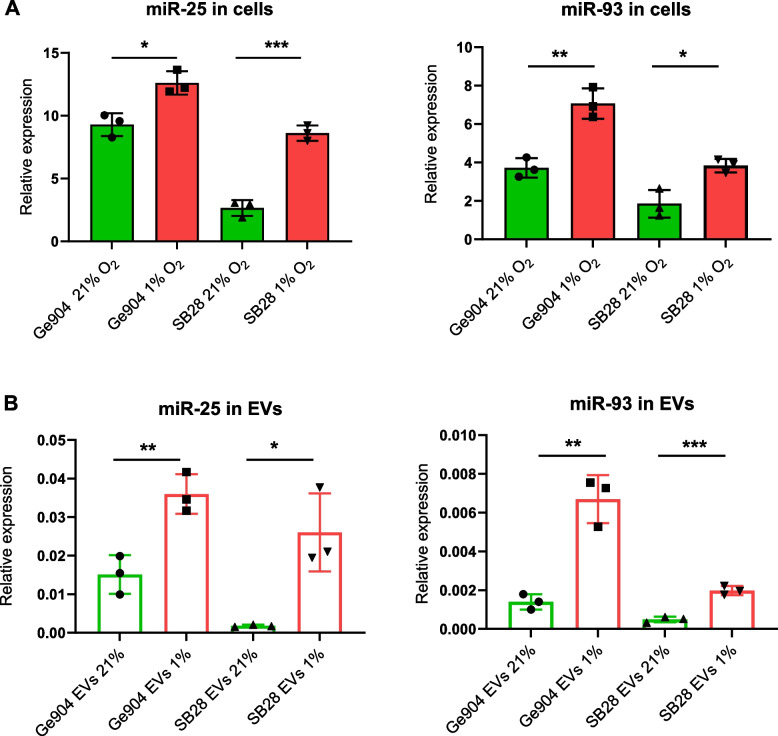
Hypoxia upregulates mir25/93 expression in human and mouse GBM cells and derived EVs. **A** Cellular expression levels of miR-25 (left) and miR-93 (right) in human (Ge904) and mouse (SB28) GBM cells measured by RT-qPCR. miR-191 was used as stably expressed housekeeping miRNA normalization control. **B** EVs secreted from human (Ge904) and mouse (SB28) GBM cells cultured for 24 h in hypoxic (1% O_2_) or normoxic (21% O_2_) conditions were analyzed for miR-25 (left) and miR-93(right) levels by RT-qPCR. cel-miR-39 spike-in control was added as a normalization control. Data is presented as the mean ± SD of three independent experiments and comparisons were made using an unpaired t test. *p < 0.05, **p < 0.005, ***P < 0.001

Hypoxia-responsive miRNAs regulate a complex spectrum of candidate target genes, including those involved in proliferation, apoptosis, metabolism and migration. Among these, it was shown that miR-25 and miR-93 are critical factors in promoting immune escape in breast cancer [27], but this has not been reported in GBM. To further investigate whether hypoxia has a direct effect on miR-25/93 expression in GBM cells and subsequently on the EVs they produce, we measured miR-25 and miR-93 content of human Ge904 and mouse SB28 GBM cell lines and of EVs released into culture by these cells. Culture in hypoxic conditions significantly increased cellular expression of miR-25 and miR-93 (Fig. 4A) compared to culture in normoxic conditions. Moreover, miR-25 and miR-93 content was higher in EVs produced by hypoxic cells (Fig. 4B). Similar results were obtained in four additional human GBM cell and corresponding EVs (Supplementary Fig. 4), with all cell lines showing upregulation of either miR-25 or miR-93 under hypoxia; in three out of four lines either miR-25 or miR-93 was exported to the EVs they secreted under hypoxia. Overall, these results indicate that hypoxia upregulates expression of miR-25/93 in both human and mouse GBM cells and their EVs.

### Hypoxic GBM-derived EVs transfer miR-25/93 to macrophages

**Fig. 5:**
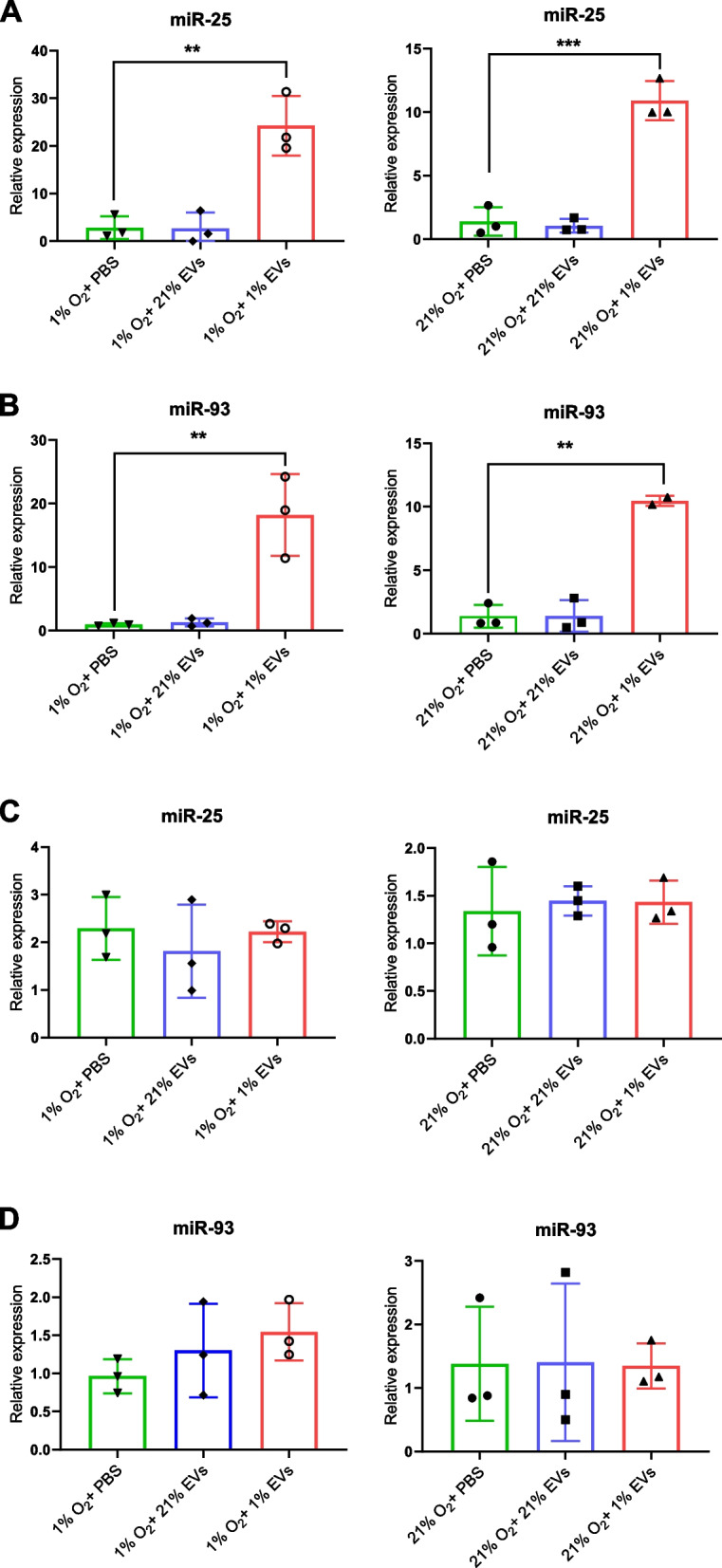
Hypoxic GBM cells deliver miR-25/93 to macrophages via hypoxic GBM-derived EVs. A., B. BMDMs were incubated for 24 h under hypoxia (1% O_2_) or normoxia (21% O_2_) with addition of EVs derived from hypoxic (1% EVs) or normoxic (21% EVs) SB28 cells. Total miRNA was extracted from the BMDMs and the levels of miR-25 (**A**) and miR-93 (**B**) were measured by RT-qPCR. C., D. BMDMs were incubated for 24 h under hypoxia (1% O_2_) or normoxia (21% O_2_) with addition of EVs derived from hypoxic (1% EVs) or normoxic (21% EVs) miR25/93 KO SB28 cells. Total miRNA was extracted from the BMDMs and the levels of miR-25 (**C**) and miR-93 (**D**) were measured by RT-qPCR. Values are expressed as mean ± SD of three biologic replicates, and comparisons were made using an unpaired t test. **p < 0.005, ***P < 0.001

We next investigated whether the GBM-derived hypoxic EVs taken up by macrophages were able to transfer their miR-25/93 cargo. We co-cultured BMDMs in normoxic and hypoxic conditions with hypoxic or normoxic SB28-derived EVs (Fig. 5). Regardless of oxygen culturing conditions, we did not observe endogenous upregulation of miR-25 or miR-93 in macrophages in the absence of EVs (Fig. 5A and B). Addition of normoxic GBM-derived EVs did not significantly change the levels of miR-25 or miR-93 in macrophages. However, when the macrophages were cultured with EVs from hypoxic SB28 cells, we observed a substantial increase of macrophage cellular levels of both miR-25/93, suggesting EV uptake and miRNA cargo delivery. Notably, this effect required miR-25/93 expression by donor cells, as EVs derived from miR-25/93 deficient SB28 cells (double KO for miR-25 and miR-93) did not result in accumulation of miR-25 or miR-93 in the cultured macrophages, even when the EVs were generated under hypoxia (Fig. 5C and D). Overall, these results indicate that hypoxia did not directly impact miR25/93 expression in macrophages and the increased levels of these miRNAs were due to the transfer of miR-25/93-containing EVs released from GBM cells under hypoxia.

### Hypoxic GBM-derived EVs dysregulate the cGAS-STING pathway resulting in downregulation of type I IFN mRNA and protein

**Fig. 6:**
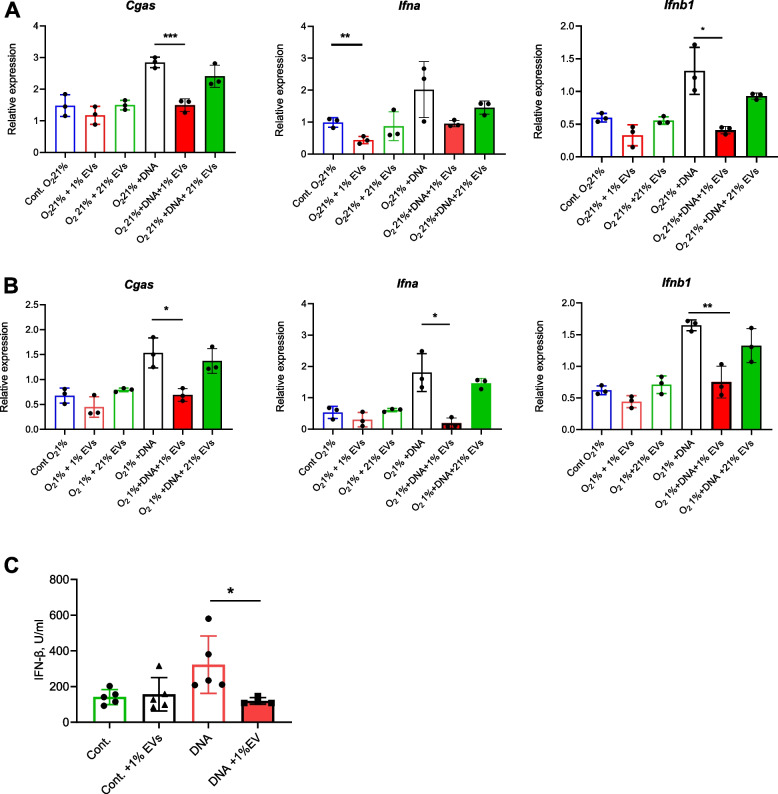
Hypoxic-GBM EVs reduce cGAS and downstream type I IFN responses in macrophages. **A**, **B** mRNA expression in EPMs of genes involved in the regulation (cGAS) and production of IFN-α and IFN-β. Macrophages were co-cultured with or without hypoxic SB28-derived EVs (1% EVs) or normoxic SB28-derived EVs (21% EVs) and 5 μg/ml of total SB28 DNA (DNA) under normoxic (O_2_ 21%) or hypoxic (O_2_ 1%) conditions for 24 h. **C** Secretion levels of IFN-β protein from EPM supernatants analyzed by ELISA. Macrophages were co-cultured with or without 5 μg/ml SB28 total DNA (DNA) and EVs from hypoxic SB28 cells (1% EVs) for 24 h. Values are expressed as mean ± SD of three biologic replicates, and comparisons were made using an unpaired t test. *p < 0.05, **p < 0.005, ***P < 0.001

To investigate the effect of miR-25/93 on cGAS-STING pathway that occurs in hypoxic EV-exposed macrophages, we used in vivo activated EPMs cultured in normoxic (21% O_2_) and hypoxic (1% O_2_) conditions either transfected with miR-25 or cultured in the presence of hypoxic SB28-derived EVs, then challenged them with DNA to activate the cGAS-STING pathway. We assessed activation of the cGAS-STING pathway by measuring cGAS, IFN-α and IFN-β mRNA expression. Transfection with miR-25 mimic, a chemically modified double-stranded RNA molecule designed to mimic specific endogenous miRNAs, inhibited cGAS and type I IFN mRNA production in DNA-challenged macrophages (Supplementary Fig. 3). cGAS and type I IFN mRNAs were downregulated in macrophages exposed to hypoxic EVs and challenged with DNA in both normoxic (Fig. 6A) and hypoxic (Fig. 6B) culture conditions. This inhibitory effect on cGAS and type I IFN mRNA expression was not observed in the conditions where macrophages were cultured with normoxic EVs. Moreover, we also observed reduction of IFN-β protein secretion (Fig. 6C), supporting the functional importance of EV-mediated inhibition of the cGAS-STING pathway.

### Hypoxic GBM-derived EVs reduce M1 associated gene expression and functions in macrophages

**Fig. 7:**
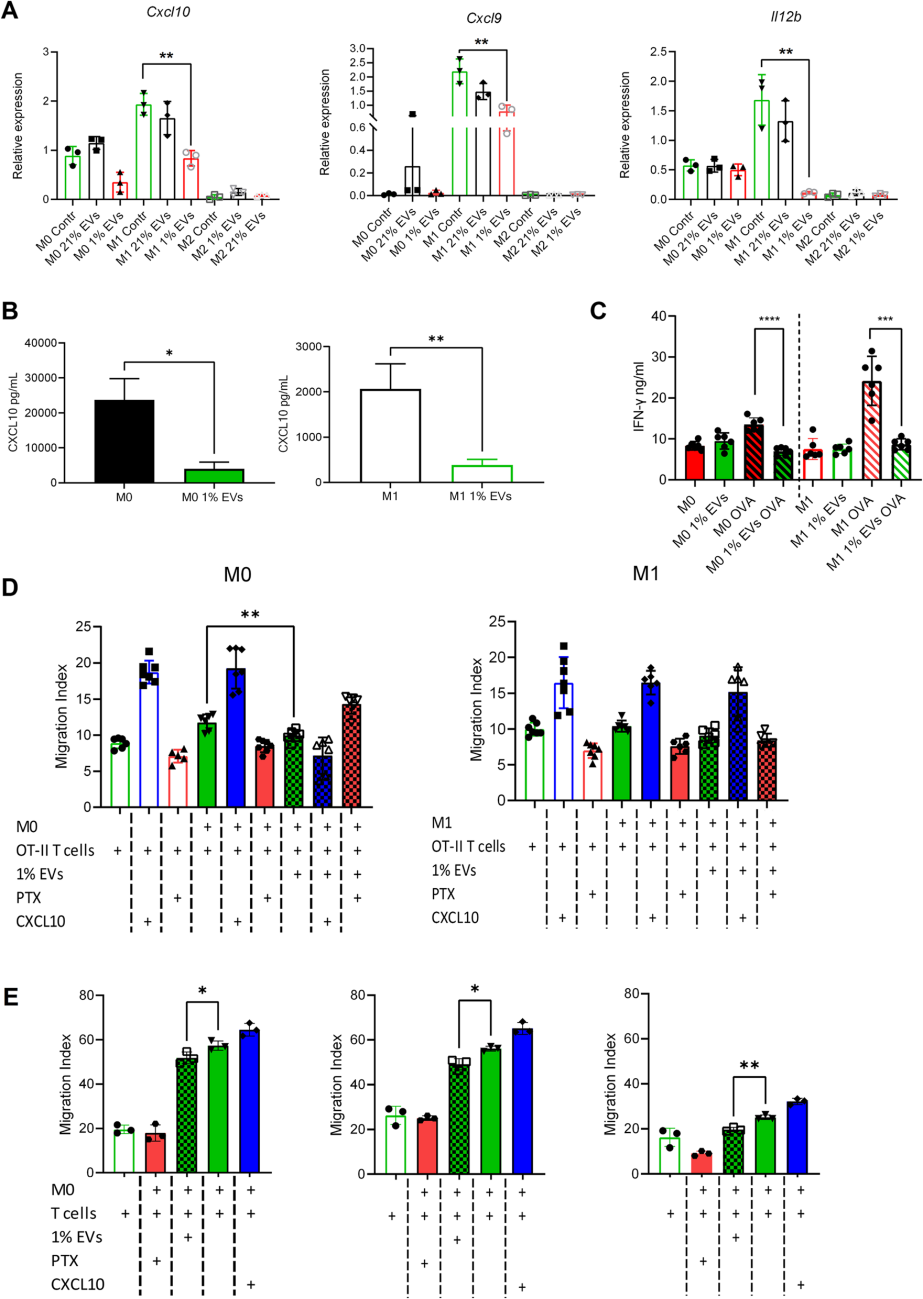
Hypoxic GBM-derived EVs disrupt T cell attraction and reactivation capacity of macrophages. **A**-**D** Mouse BMDMs were unpolarized (M0), M1 polarized (M1), or M2 polarized (M2) for 7 days in the absence (contr.) or presence of hypoxic GBM-derived EVs (1% EVs) or normoxic GBM-derived EVs (21% EVs). **A** mRNA expression of *Cxcl9, Cxcl10* and *Il12b* in BMDMs. mRNA levels were measured by RT-qPCR and expression was normalized to housekeeping genes (G*apdh and Eef1a1*). **B** Macrophages were harvested, washed, then incubated for a further 24 h. Supernatants were collected and CXCL10 secretion was measured by a flow cytometry bead-based assay. **C** Macrophages were harvested, washed, pulsed with OVA peptide then added to OT-II CD4^+^ cells. IFN-γ secretion was measured after 24 h by ELISA. **D** (Left panel) Macrophages were harvested, washed, then added to the lower chambers of Transwell plates for 3 h. CD4^+^ OT-II T cells were added to the upper chamber and migration measured after 6 h. Negative migration control: CD4^+^ T cells were pretreated with pertussis toxin (PTX). Positive migration control: CXCL10 was added to the BMDMs in the lower chamber (CXCL10). **E** (Left, center and right panel) Human MDMs (M0) from three donors were harvested, washed, then added to the lower chambers of Transwell plates for 3 h. (Left and center) CD3^+^ T cells from the corresponding buffy coat donor were added to the upper chamber and the migration was measured after 6 h. Negative migration control: CD3^+^ T cells were pretreated with pertussis toxin (PTX). Positive migration control: CXCL10 was added to the human macrophages in the lower chamber. (Right panel) Identical experiment as in left and center panels with the use of CD3^+^ T cells from a different donor. Values are expressed as mean ± SD of three biologic replicates, and comparisons were made using an unpaired t test. *p < 0.05, **p < 0.005, ***P < 0.001, ****P < 0.0001

In view of the well-established link between M2-polarized TAMs and tumor progression, we explored in vitro whether EVs from hypoxic GBM cells might play a role in the process of polarizing newly tumor infiltrating M0 macrophages through inhibition of cGAS and reduction of the cGAS-STING type I interferon response. We cultured non-polarized (M0) BMDMs in the presence of hypoxic or normoxic SB28-derived EVs, and either M1 or M2 polarizing stimuli. As expected, without addition of EVs, all the genes associated with M1 or M2 polarization were overexpressed in the corresponding conditions (Supplementary Fig. 6). However, the addition of hypoxic SB28-derived EVs resulted in significantly reduced *Cxcl9*, *Cxcl10* and *Il12b* gene expression in M1 macrophages (Fig. 7A). mRNA expression of other tested genes was not significantly reduced (Supplementary Fig. 6). Notably, using human hypoxic GBM EV preparations and human MDMs we also saw downregulation of *CXCL9*, *CXCL10* and *IL12B* with EVs from Ge904 and *CXCL10* downregulation with EVs from Ge835 (Supplementary Fig. 5). These cell lines have a distinct profile of miR-25/93 expression under both normoxic and hypoxic conditions. Ge835 expresses lower levels of miR-25/93 and Ge904 expresses higher levels of miR25/93 which is reflected in the miR cargo of the EVs they secrete. Hypoxia induces the expression of miR-25/93 in both cell lines and the effect of their EVs is different on the recipient macrophages, with Ge835 having a milder effect on the inhibition of the *CXCL9, CXCL10* and *IL12 *genes.

**Fig. 8:**
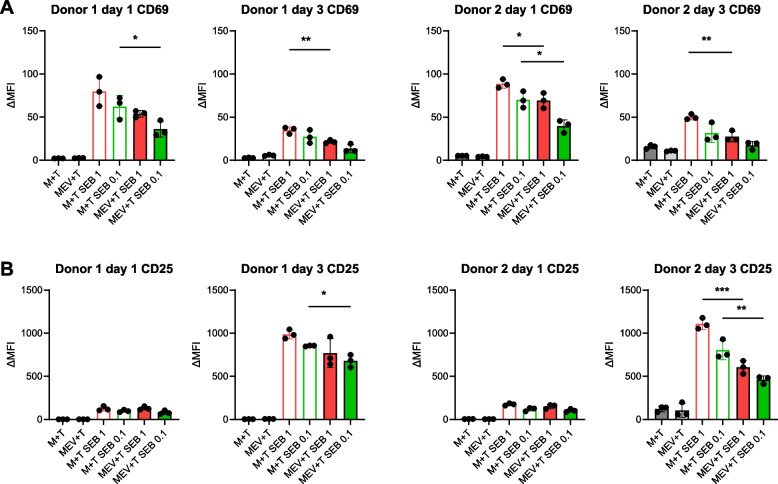
Human macrophages treated with hypoxic GBM-derived EVs have lower capacity to induce T cell activation markers after superantigen stimulation. A., B. Human MDMs from two donors (Donor 1 and Donor 2) were cultured for 7 days in the absence (M) or presence (MEV) of hypoxic GBM-derived EVs (1% EVs). The macrophages were then washed and co-cultured with CD3^+^ T cells from the same donor for 3 days with or without the addition of Staphylococcal Enterotoxin B (1 μg/ml, SEB 1 or 0.1 μg/ml, SEB 0.1). On day 1 and day 3 cells were harvested and the expression of (**A**) CD69 and (**B**) CD25 on T cells (CD4^+^ and CD8^+^) was measured by flow cytometry. Values are expressed as mean ± SD of three replicates for each donor, and comparisons were made using an unpaired t test. *p < 0.05, **p < 0.005, ***P < 0.001

For mouse BMDMs we also tested secreted chemokine; we detected significantly lower levels of CXCL10 secreted by hypoxic EV-treated M0 and M1 BMDMs as compared to PBS-treated BMDM (Fig. 7B). These results establish a functionally important consequence of cGAS-STING pathway downregulation by hypoxic GBM EVs, namely reduced mRNA expression of the CD4^+^ Th1 T cell and CD8^+^ cytotoxic T cell promoting cytokine *IL12b,* and T-cell recruiting chemokines *Cxcl9/Cxcl10.* We next determined if hypoxic GBM-derived EVs used to treat BMDMs could alter macrophage capacity to efficiently attract and induce IFN-γ production in CD4^+^ T cells by performing a co-culture assay. To test this, we used M0 or M1 BMDMs cultured with or without hypoxic SB28-derived EVs during the polarization. We used previously activated OT-II CD4^+^ T cells as responders. After 24 h, OT-II CD4^+^ T cells were reactivated by OVA-peptide pulsed M0 and M1 macrophages. When macrophages were polarized in the presence of hypoxic EVs they could not efficiently reactivate OVA specific CD4+ T cells, resulting in low IFN-γ production (Fig. 7C). CXCL10 is normally produced by macrophages to recruit activated CXCR3^+^ T cells, thus we evaluated whether hypoxic EV-treated M0 and M1 BMDMs had impaired capacity to attract CD4^+^ T cells using a transwell migration assay. As expected, exogenous CXCL10 promoted CD4^+^ T cell migration whilst pertussis toxin pretreated CD4^+^ T cells (to inhibit GPCR signaling of chemokine receptors) had impaired migration. However, hypoxic EV-treated M0 BMDMs could not induce the same migration capacity in CD4^+^ T cells compared with PBS treated cells (Fig. 7D and Supplementary Fig. 8A). Regarding human macrophage function after exposure to EVs from human hypoxic GBM cells, we tested chemoattraction of T cells as well as T cell activation after superantigen or alloantigen stimulation. Human T cell migration towards macrophages was significantly inhibited when macrophages had been exposed to hypoxic GBM-derived EVs (Fig. 7E and Supplementary Fig. 8A). Human T cell activation was tested by superantigen induced upregulation of CD69 and CD25; this was significantly inhibited under some conditions (time point or superantigen concentration, according to the experiment) when macrophages from two different donors were exposed to hypoxic GBM-derived EVs (Fig. 8A, B). Moreover, supernatants collected from day 3 superantigen stimulated cells from the 2 donors showed a trend for lower IFN-γ content after hypoxic GBM-derived EV exposure, although this did not always reach statistical significance (data not shown). Human T cell activation after alloantigen stimulation using different allogeneic combinations of macrophages and T cells was also significantly inhibited in most cultures, based on reduced CD69 and CD25 expression when the macrophages had been pre-treated with hypoxic GBM-derived EVs (Supplementary Fig. 7). Overall, these experiments indicate that hypoxic GBM-derived EVs prevented efficient macrophage polarization towards an M1-like status, as measured by reduced expression of key M1-associated genes. The functional consequences of this were that we measured lower T cell chemoattraction and CD4^+^ T cell reactivation (in mouse T cells) or superantigen/alloantigen activation (in human T cells) by the EV-treated macrophages.

## Discussion

The immunosuppressive potential of cancer-derived EVs has been described in various tumor malignancies, with compelling data showing EV participation in TME formation, tumor progression and modulation of immune responses [14, 58, 59]. However, the direct and indirect effects of GBM-derived EVs on myeloid cells are still poorly described. In GBM, hypoxia is an important feature of the TME that has been shown to increase the production of EVs and to alter their cargos. Here, we show that hypoxia induced EV secretion in cells from multiple early passage human GBM lines as well as in established human and mouse GBM cell lines. These results extend previous findings in established GBM cell lines (U87, U251 and C3) [60] and in breast cancer [61] where it was observed that hypoxia induced EV secretion. Furthermore, we observed that hypoxia stimulated the expression of miR-25/93 in GBM cells and that these cells secreted EVs containing elevated levels of miR-25/93. Our data highlight the role of hypoxia in driving release of miR-25/93 containing EVs, which might be an underlying mechanism responsible for the accumulation of miR-25/93 in EVs previously reported in a range of solid cancers including glioma [23, 62–65]. Our in vitro findings suggest that the export of miR-25/93 in hypoxic EVs occurs in many GBM cell lines despite previously reported heterogeneous hypoxia responses in GBM [2]. The characterization of the EVs from human and murine cell lines used in this study showed that the GBM cells are secreting heterogeneous EV populations consisting of exosomal and microvesicle fractions. However, all of the cell lines are upregulating the EV secretion under hypoxia and these EVs are carrying specific EV associated markers. Notably, despite their heterogenous nature, each of the GBM cell lines responded to hypoxia by upregulating miR-25/93, which was also reflected in the EVs they produced. We also tested human GBM biopsies for miR-25/93 expression, which were positive in 5/5 tumors tested by qPCR (data not shown). These data, together with our observations of elevated miR-25/93 expression in GBM cells and EVs would be consistent with the upregulated miR-25/93 expression observed in ischemic non-malignant brain [66], and also in the hypoxic regions of breast cancer [27], although these studies did not report miR-25/93 in the context of EVs.

The ability of GBM cells to produce high levels of EVs under hypoxia and the overexpression of miR-25/93 that are exported in hypoxic EVs has been mostly studied in the context of uptake by other cancer cells, however, they could have significant effects on stromal cells if they are actually taken up by these cells and if they modulate their function. Since the most abundant non-malignant cells in the GBM microenvironment are the myeloid cells, we focused on macrophages, which readily acquired hypoxic miR-25/93 containing EVs in vitro, consistent with a previous report in which the transfer of a different miRNA cargo was reported [67]. It is important to note that EV uptake is not necessarily sufficient to induce functional changes, since internalized EVs do not always release their cargo into the cytosol [68]. However, our results indicate that that this was not the case for the macrophages that we tested. Of particular note, although macrophages have been reported to upregulate miR-25 expression in the context of infection [69], we did not observe hypoxia-promoted induction of endogenous miR-25/93 expression. This suggests that although macrophages might be resistant to the direct immunomodulatory effects of hypoxia-induced miR-25/93, they can still be indirectly affected through uptake of hypoxic GBM cell-derived miR25/93.

The endogenous stimulation of the cGAS-STING pathway in cancer can occur through uptake of tumor cell derived DNA, which will be abundant in hypoxic and necrotic zones that promote DNA damage and nuclear leaks [32]. The anti-tumor potential of robust cGAS-STING pathway activation in GBM has been demonstrated by therapeutic use of the synthetic STING agonist cGAMP that promotes innate and adaptive anti-tumor immunity [33, 70, 71]. This suggests that the cGAS-STING pathway in macrophages infiltrating the TME is kept functionally active but repressed due to factors in the TME that inhibit or repress key points along the cGAS-STING-type I IFN axis. We demonstrate that exposure of GBM cells to hypoxia induces miR-25/93-containing EVs that can be transferred to macrophages, resulting in reduced cGAS expression, and downregulation of type I IFN mRNA and IFN-β protein secretion. Indeed, cGAS-STING pathway induction was shown to be crucial for reprograming M2-like pro-tumoral macrophages into an M1-like anti-tumoral state in BMDMs and in ex vivo TAMs from colorectal and breast cancer [72, 73]. Our mRNA expression analysis indicated that macrophages exhibit inefficient M1 polarization in the presence of hypoxic GBM-derived EVs; specifically, they downregulate mRNA for the chemokines *Cxcl9 Cxcl10* as well as for *Il12b*, which are typically expressed by M1-like macrophages. CXCL9 and CXCL10 play an important role in T cell attraction and *Il12*b is expressed by activated macrophages that favor Th1 differentiation and functions such as IFN-γ production by effector CD4^+^ and CD8^+^ T cells [74–76]. CXCL9 and CXCL10, also known as monokines induced by gamma interferon (MIG), are mainly induced by IFN-γ [77]. However, CXCL9 and CXCL10 expression can be induced in response to type I IFN [78], expression of which can be directly induced by cGAS-STING pathway activation. Indeed, it was shown that KO of STING in mice resulted in complete elimination of *Cxcl10* mRNA expression in BMDMs, highlighting the key role of STING in regulating CXCL10 production [79]. Moreover, activation of the cGAS-STING signal pathway in microglia induces the expression of pro-inflammatory cytokines including IL-12 [80]. Our findings highlight how a prominent feature of the tumor microenvironment, hypoxia, can perturb this essential cGAS-STING axis in immune cells, through hypoxic GBM-derived EVs repressing cGAS expression in macrophages, thereby inhibiting cGAS-STING pathway activation and type I IFN production, with direct consequences for anti-tumor immunity mediated by T cells. Interestingly we observed that *Il12b* expression was also impaired in macrophages exposed to hypoxic GBM-derived EVs. In adenocarcinoma, STING activation by cGAMP induced *IL12* expression in tumor tissues [81] and IL-12 levels in response to DNA were reduced in STING- or cGAS-deficient dendritic cells [82]. Thus, the inhibition of the cGAS-STING pathway in macrophages by hypoxic GBM EVs with their miR-25/93 cargo elucidates an underlying mechanism for impaired anti-tumor immune functions seen in hypoxic GBM tumors [83].

Based on our in vitro data, we suggest that in the TME of GBM, cancer cells in hypoxic regions of the tumor have the potential to modulate the anti-tumor properties of recruited and resident macrophages. Mechanistically, we propose that this will occur by hypoxia-induced overexpression of miR-25/93 and enhanced secretion of EVs containing these miRs as cargo, which will subsequently be taken up by the abundant infiltrating macrophages in the TME. This could lead to miR-25/93-mediated inhibition of cGAS and type I IFN production, and influence macrophage functions by down regulating M1-associated genes, cytokine production and T-cell reactivation capacities.

## Conclusions

GBM cancer cells release EVs, a process that is augmented under hypoxic conditions. The elevated numbers of hypoxia-induced EVs also carry a potent hypoxia-shaped miRNA cargo that negative impacts recipient macrophages. In this study, using human and mouse origin GBM cells, macrophages and T cells, we demonstrate potentially immunosuppressive consequences of hypoxia-induced EVs on key cells that determine the pro-or anti-tumoral immune status in the tumor bed. Adequate activation of the cGAS-STING pathway and expression of M1-associated genes in macrophages are central to efficacious anti-tumor immunity, but they are compromised in the presence of miR-25/93 that can be imported by EVs released by hypoxic GBM cells. This mechanism can reinforce and extend immunosuppression from hypoxic regions to more globally shape the TME.

### Supplementary Information

**Additional file 1: Supplementary Fig. 1.** TEM images of EVs secreted by human (Ge835 and LN18) and murine (GL261) GBM cell lines. Pictures are representative of at least 6 images.

**Additional file 2: Supplementary Fig. 2.** BODIPY staining of macrophages. BODIPY at a concentration of 1 mM was added to macrophages cultured in removable silicone chambers on a glass slide for 12 h. After that macrophages were fixed, stained and imaged. A. Human MDMs stained for CD68 and DAPI B. Murine BMDMs stained for F4/80 and DAPI.

**Additional file 3: Supplementary Fig. 3.** miR-25 transfection inhibits cGAS, IFN-α, and IFN-β gene expression in macrophages. EPMs were transfected with miRNA 25 mimic (miR-25) and challenged with 5 μg/ml of total SB28 DNA (DNA). The expression of IFN-α, IFN-β and cGAS after 24 h was measured by RT-qPCR. Values are expressed as mean ± SD of three biologic replicates, and comparisons were made using an unpaired t test. *p < 0.05, **p < 0.005, ***P < 0.001.

**Additional file 4: Supplementary Fig. 4.** Hypoxia increases EV secretion and upregulates mir25/93 expression in cells and corresponding EVs from human GBM cell lines. A. NTA profiles of EVs isolated from human GBM cell lines Ge738, Ge982 and Ge975 cultured in hypoxic (1% O2) or normoxic (21% O2) conditions. EV depleted culture medium was used as control. The calculated size distribution is depicted as a mean from three experiments and three measurements. B. Cellular expression levels of miR-25 (top left) and miR-93 (top right) in human GBM cell lines Ge738, Ge982, Ge975 and Ge835 GBM cells measured by RT-qPCR. miR-191 was used as stably expressed housekeeping miRNA as a normalization control. EVs secreted from human Ge738, Ge982, Ge975 and Ge835 GBM cells cultured for 24 h in hypoxic (1% O_2_) or normoxic (21% O_2_) conditions were analyzed for miR-25 (bottom left) and miR-93 (bottom right) levels by RT-qPCR. cel-miR-39 spike-in control was added as a normalization control. Values are expressed as mean ± SD of three biologic replicates, and comparisons were made using an unpaired t test. *p < 0.05, **p < 0.005, ***P < 0.001.

**Additional file 5: Supplementary Fig. 5.** mRNA expression for *CXCL9*, *CXCL10* and *IL12* in human MDMs. Human MDMs were incubated under 21% or 1% O_2_ in the absence (contr.) or presence of hypoxic GBM-derived EVs (1% EVs) or normoxic GBM-derived EVs (21% EVs) collected from two human GBM cells (A) Ge835 and (B) Ge904. The mRNA levels were measured by RT-qPCR and expression was normalized to housekeeping genes (*GAPDH* and *EEF1A1*). Values are expressed as mean ± SD of three biologic replicates, and comparisons were made using an unpaired t test. **p < 0.005, ***P < 0.001.

**Additional file 6: Supplementary Fig. 6.** Expression of genes involved in M1, M0 and M2 polarization in BMDMs. mRNA expression of 9 genes involved in polarization of macrophages: M0 (*Stab1 and Cd163*), M1 (*Ccl5, Il1b, Stat1* and *Tnfa*) and M2 (*Cdh1, Arg1* and *Mrc1*). mRNA expression of 3 genes is shown in Fig. 6. BMDMs were cultured for 7 days, either unpolarized (M0) or polarized towards M1 or M2. Cells were cultured in the presence of hypoxic (1% EVs) or normoxic (21% EVs) GBM-derived EVs or in media control. mRNA levels were detected by RT-qPCR expression was normalized to housekeeping genes (G*apdh* and E*ef1a1*).

**Additional file 7: Supplementary Fig. 7.** Human macrophages treated with hypoxic GBM-derived EVs have lower capacity to induce T cell activation markers after allogeneic stimulation. Human MDMs from two donors (Donor 1 and Donor 2) were cultured in the absence (M) or presence (MEV) of hypoxic GBM-derived EVs (1% EVs) for 7 days. On day 7 MDMs were cocultured with CD3^+^ T cells from three different donors (T3, T4 and T5) for 3 days. On day 1 and day 3 cells were harvested and the expression of (A) CD69 and (B) CD25 on T cells (CD4^+^ and CD8^+^) was measured by flow cytometry. Values are expressed as mean ± SD of three replicates from each MDM/T cell coculture, and comparisons were made using an unpaired t test. *p < 0.05, **p < 0.005, ***P < 0.001.

**Additional file 8: Supplementary Fig. 8.** Representative flow cytometry readout for T cell migration assays shown in Fig. 7D, E. (A) Flow cytometry graph showing migration of murine CD4^+^ T cells from lower Transwell chamber in MO (control) and M0 + 1% EV conditions. (B) Flow cytometry graph showing migration of human CD3^+^ T cells from lower Transwell chamber in MO (control) and M0 + 1% EV conditions.

**Additional file 9: Supplementary Table 1.** Primer sequences for qRT–PCR.

## Data Availability

All data supporting the findings of this study are available within the article and the supplemental information, or from the corresponding author upon reasonable request. This study includes no data deposited in external repositories.

## Abbreviations

GBM: Glioblastoma
EV: Extracellular vesicle
TME: Tumor microenvironment
dsDNA: Double-stranded DNA
BMDM: Bone marrow derived macrophage
EPM: Elicited peritoneal macrophage
MDM: Monocyte derived macrophages
NTA: Nanoparticle tracking analysis
TAM: Tumor-associated macrophage
