# Supplementary figures and images for: Hypoxic glioblastoma-cell-derived extracellular vesicles impair cGAS-STING activity in macrophages
Source: Cell Commun Signal. 2024 Feb 22;22:144. doi: 10.1186/s12964-024-01523-y (PMC10882937; doi:10.1186/s12964-024-01523-y)

Ge835

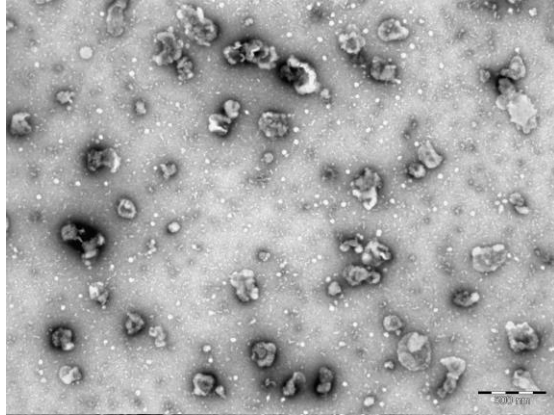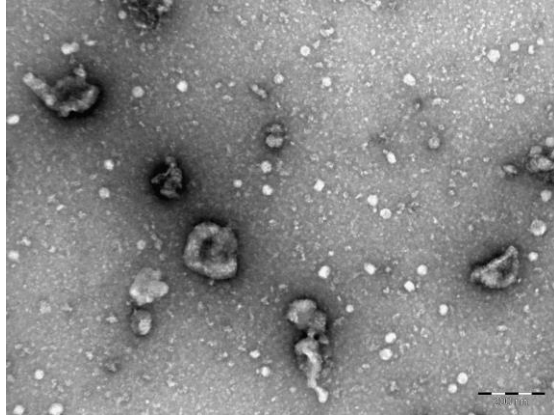

LN18

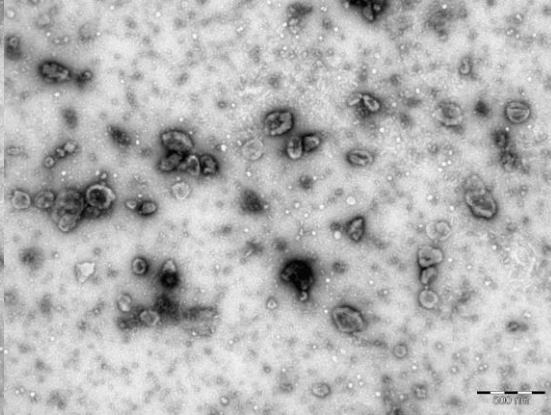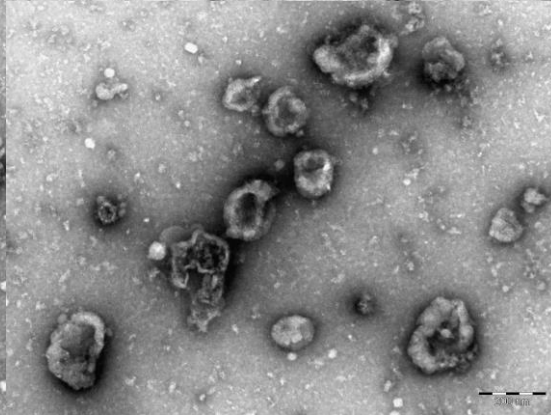

GL261

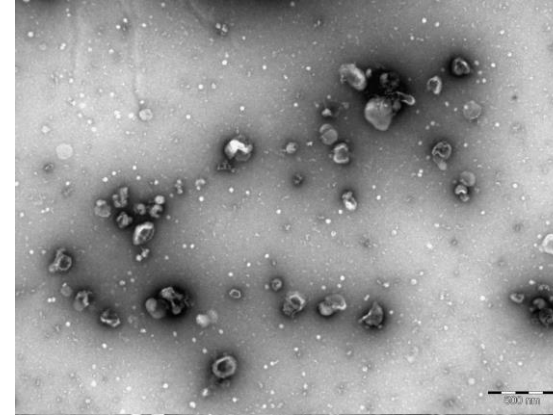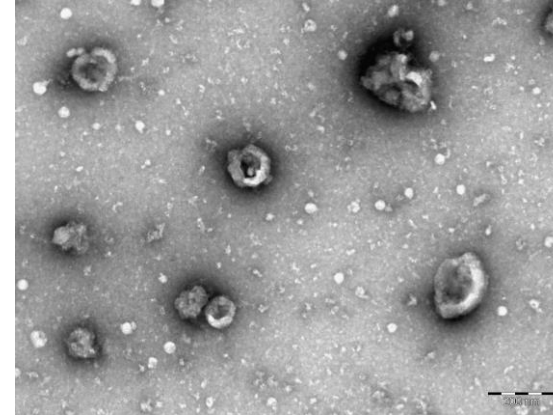

Supplement: Supplementary file 1 — Additional file 1: Supplementary Fig. 1. TEM images of EVs secreted by human (Ge835 and LN18) and murine (GL261) GBM cell lines. Pictures are representative of at least 6 images. [file 12964_2024_1523_MOESM1_ESM.pdf]

A      **DAPI**                                      **BODIPY**                                      **CD68**                                      **Merged**

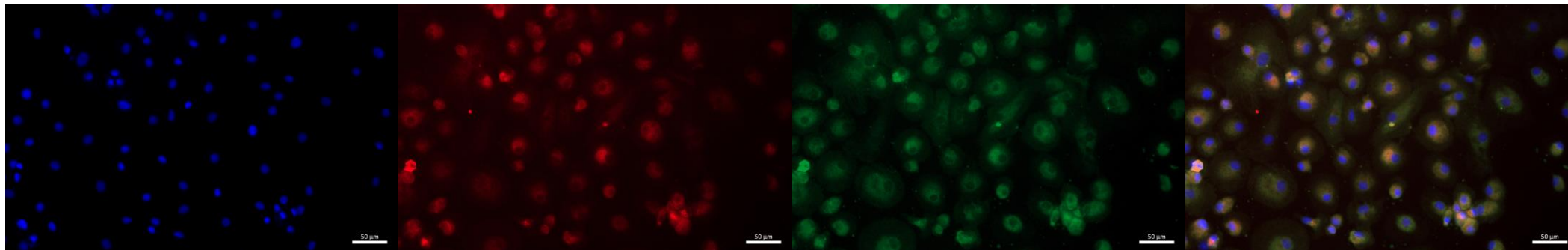

B      **DAPI**                                      **BODIPY**                                      **F4/80**                                      **Merged**

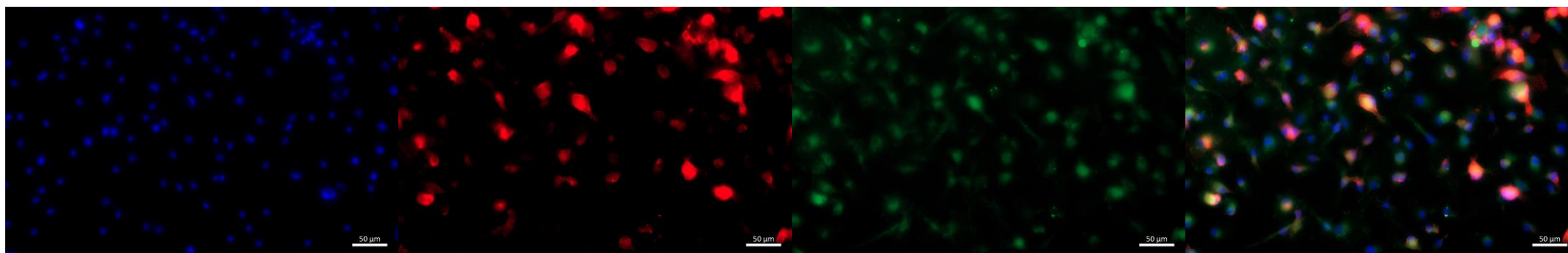

Supplement: Supplementary file 2 — Additional file 2: Supplementary Fig. 2. BODIPY staining of macrophages. BODIPY at a concentration of 1 mM was added to macrophages cultured in removable silicone chambers on a glass slide for 12 h. After that macrophages were fixed, stained and imaged. A. Human MDMs stained for CD68 and DAPI B. Murine BMDMs stained for F4/80 and DAPI. [file 12964_2024_1523_MOESM2_ESM.pdf]

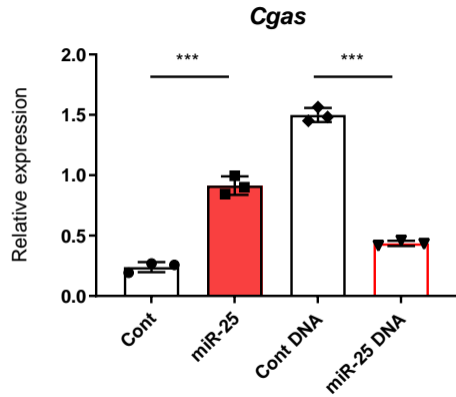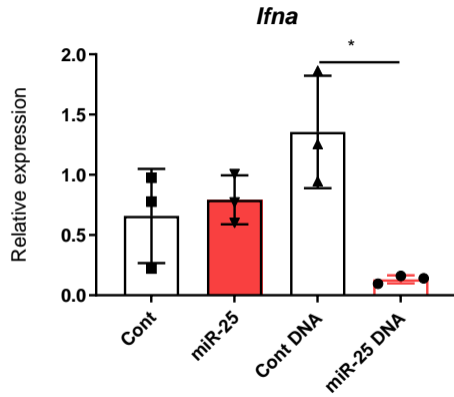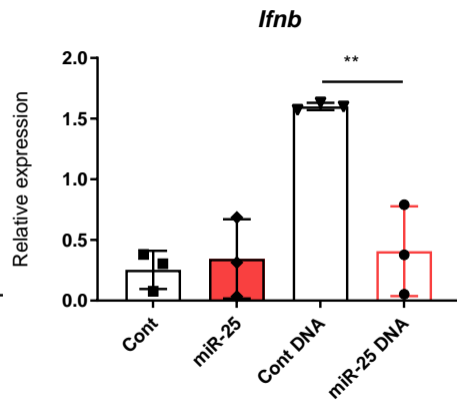

Supplement: Supplementary file 3 — Additional file 3: Supplementary Fig. 3. miR-25 transfection inhibits cGAS, IFN-α, and IFN-β gene expression in macrophages. EPMs were transfected with miRNA 25 mimic (miR-25) and challenged with 5 μg/ml of total SB28 DNA (DNA). The expression of IFN-α, IFN-β and cGAS after 24 h was measured by RT-qPCR. Values are expressed as mean ± SD of three biologic replicates, and comparisons were made using an unpaired t test. *p < 0.05, **p < 0.005, ***P < 0.001. [file 12964_2024_1523_MOESM3_ESM.pdf]

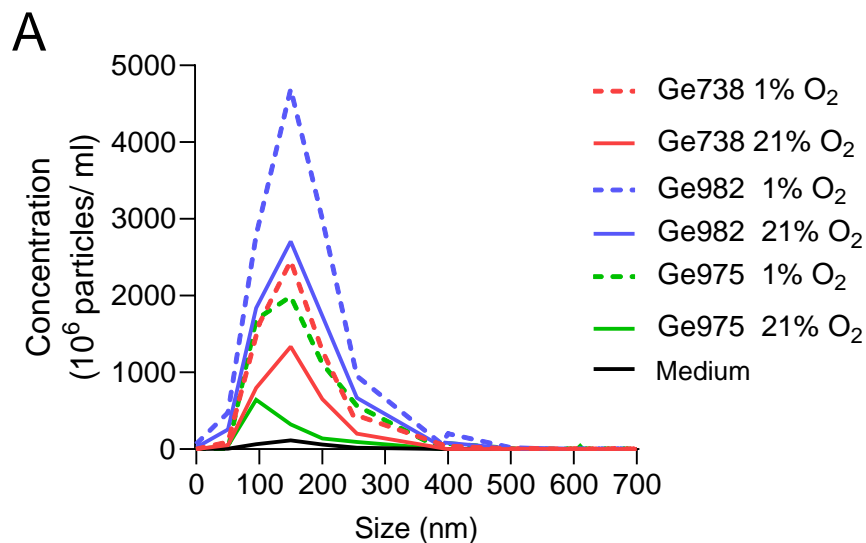

**B**

**miR-25 in cells**

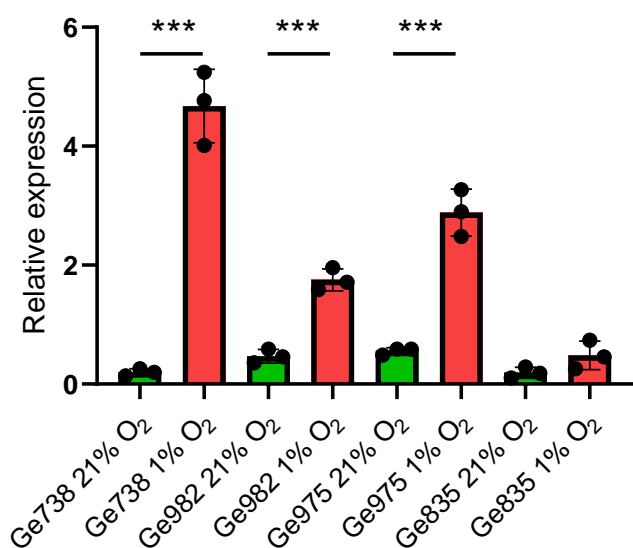

**miR-93 in cells**

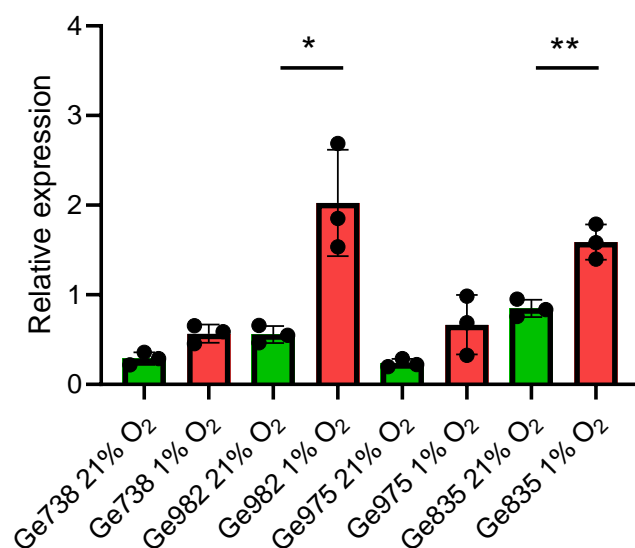

**miR-25 in EVs**

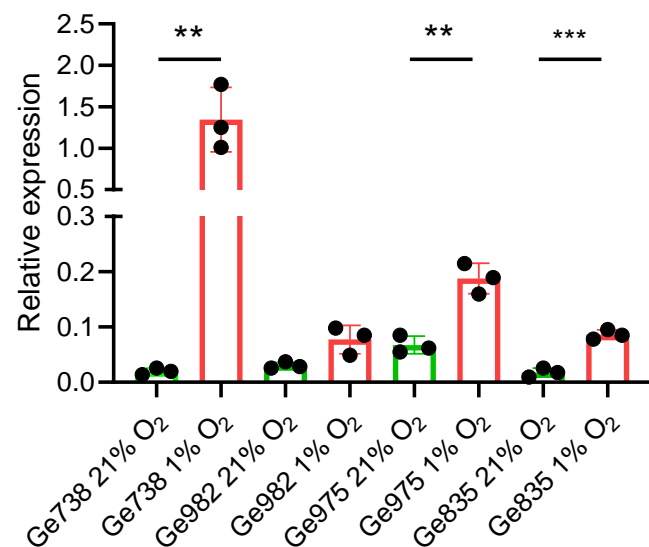

**miR-93 in EVs**

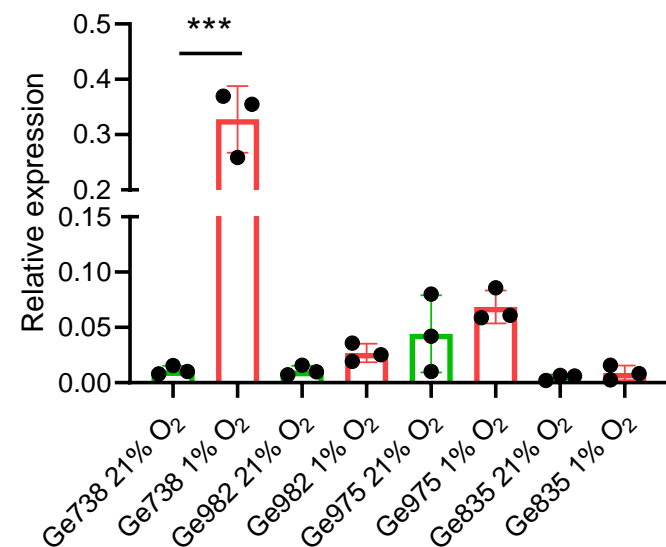

Supplement: Supplementary file 4 — Additional file 4: Supplementary Fig. 4. Hypoxia increases EV secretion and upregulates mir25/93 expression in cells and corresponding EVs from human GBM cell lines. A. NTA profiles of EVs isolated from human GBM cell lines Ge738, Ge982 and Ge975 cultured in hypoxic (1% O2) or normoxic (21% O2) conditions. EV depleted culture medium was used as control. The calculated size distribution is depicted as a mean from three experiments and three measurements. B. Cellular expression levels of miR-25 (top left) and miR-93 (top right) in human GBM cell lines Ge738, Ge982, Ge975 and Ge835 GBM cells measured by RT-qPCR. miR-191 was used as stably expressed housekeeping miRNA as a normalization control. EVs secreted from human Ge738, Ge982, Ge975 and Ge835 GBM cells cultured for 24 h in hypoxic (1% O2) or normoxic (21% O2) conditions were analyzed for miR-25 (bottom left) and miR-93 (bottom right) levels by RT-qPCR. cel-miR-39 spike-in control was added as a normalization control. Values are expressed as mean ± SD of three biologic replicates, and comparisons were made using an unpaired t test. *p < 0.05, **p < 0.005, ***P < 0.001. [file 12964_2024_1523_MOESM4_ESM.pdf]

A

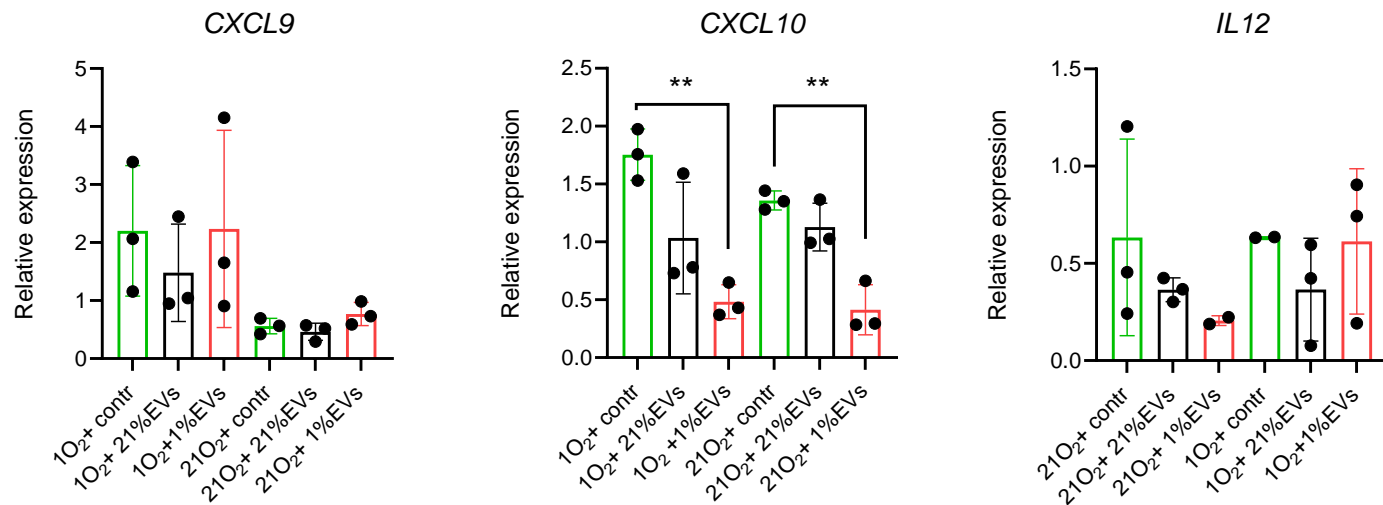

B

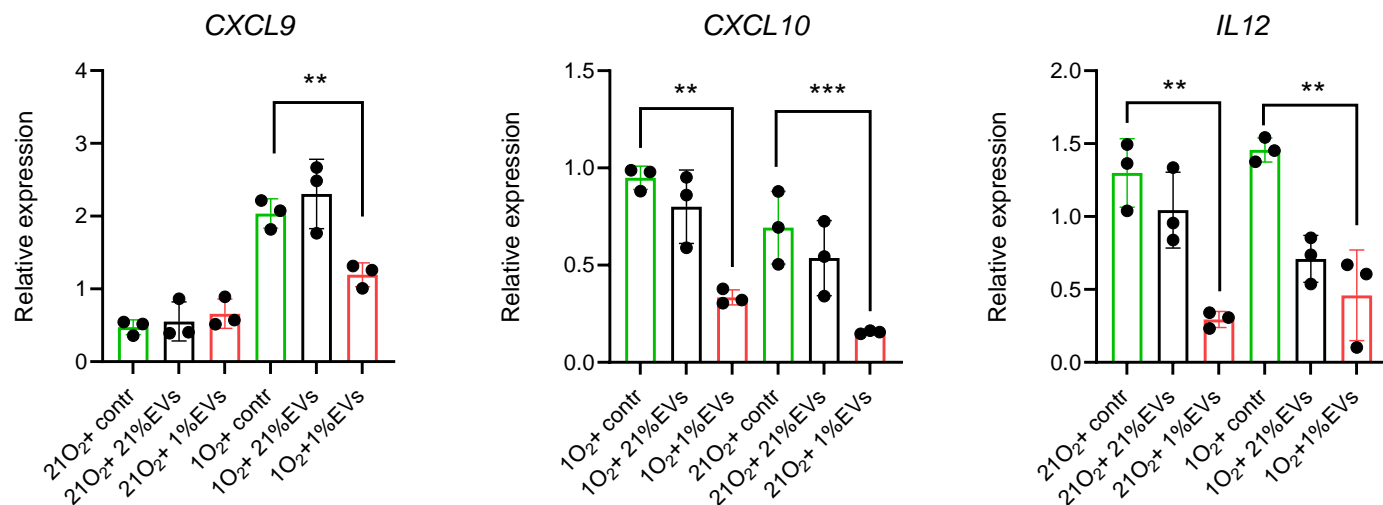

Supplement: Supplementary file 5 — Additional file 5: Supplementary Fig. 5. mRNA expression for CXCL9, CXCL10 and IL12 in human MDMs. Human MDMs were incubated under 21% or 1% O2 in the absence (contr.) or presence of hypoxic GBM-derived EVs (1% EVs) or normoxic GBM-derived EVs (21% EVs) collected from two human GBM cells (A) Ge835 and (B) Ge904. The mRNA levels were measured by RT-qPCR and expression was normalized to housekeeping genes (GAPDH and EEF1A1). Values are expressed as mean ± SD of three biologic replicates, and comparisons were made using an unpaired t test. **p < 0.005, ***P < 0.001. [file 12964_2024_1523_MOESM5_ESM.pdf]

**M0**

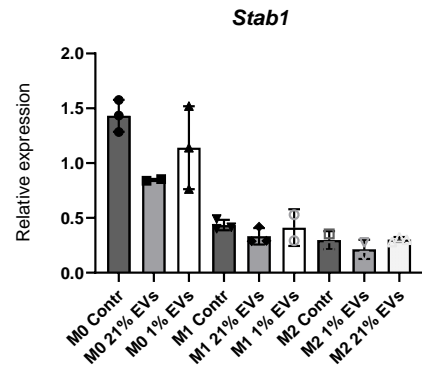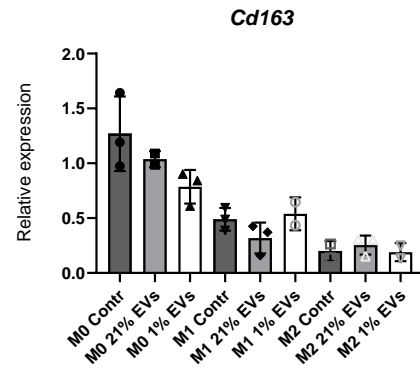

**M1**

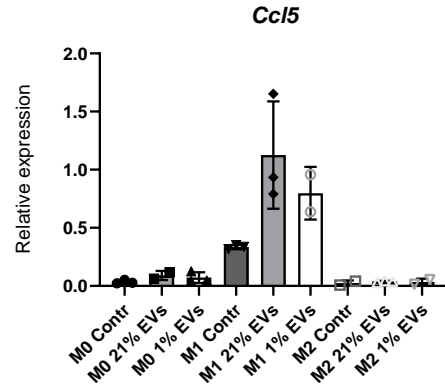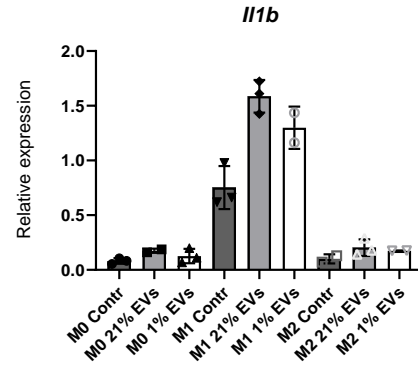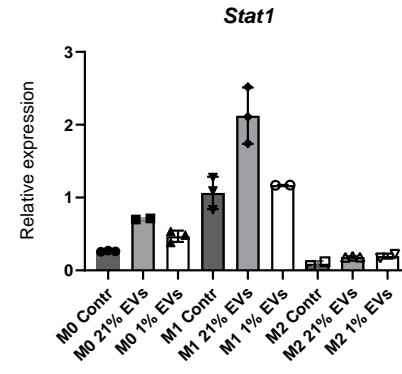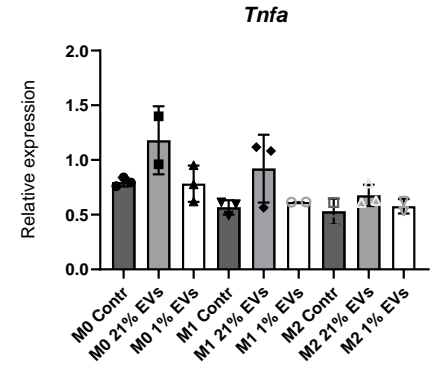

**M2**

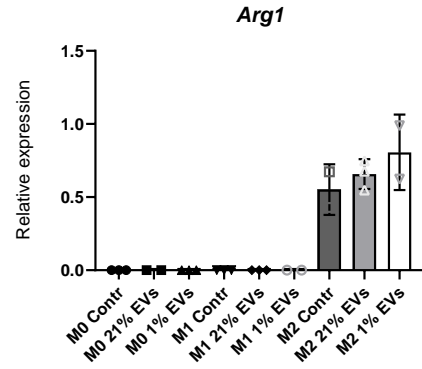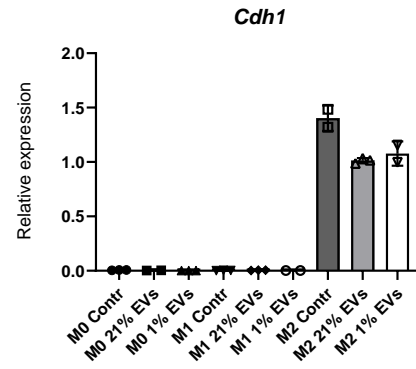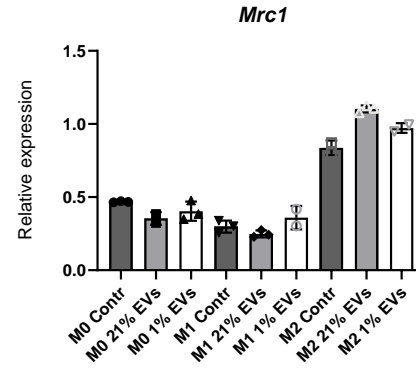

Supplement: Supplementary file 6 — Additional file 6: Supplementary Fig. 6. Expression of genes involved in M1, M0 and M2 polarization in BMDMs. mRNA expression of 9 genes involved in polarization of macrophages: M0 (Stab1 and Cd163), M1 (Ccl5, Il1b, Stat1 and Tnfa) and M2 (Cdh1, Arg1 and Mrc1). mRNA expression of 3 genes is shown in Fig. 6. BMDMs were cultured for 7 days, either unpolarized (M0) or polarized towards M1 or M2. Cells were cultured in the presence of hypoxic (1% EVs) or normoxic (21% EVs) GBM-derived EVs or in media control. mRNA levels were detected by RT-qPCR expression was normalized to housekeeping genes (Gapdh and Eef1a1). [file 12964_2024_1523_MOESM6_ESM.pdf]

**A**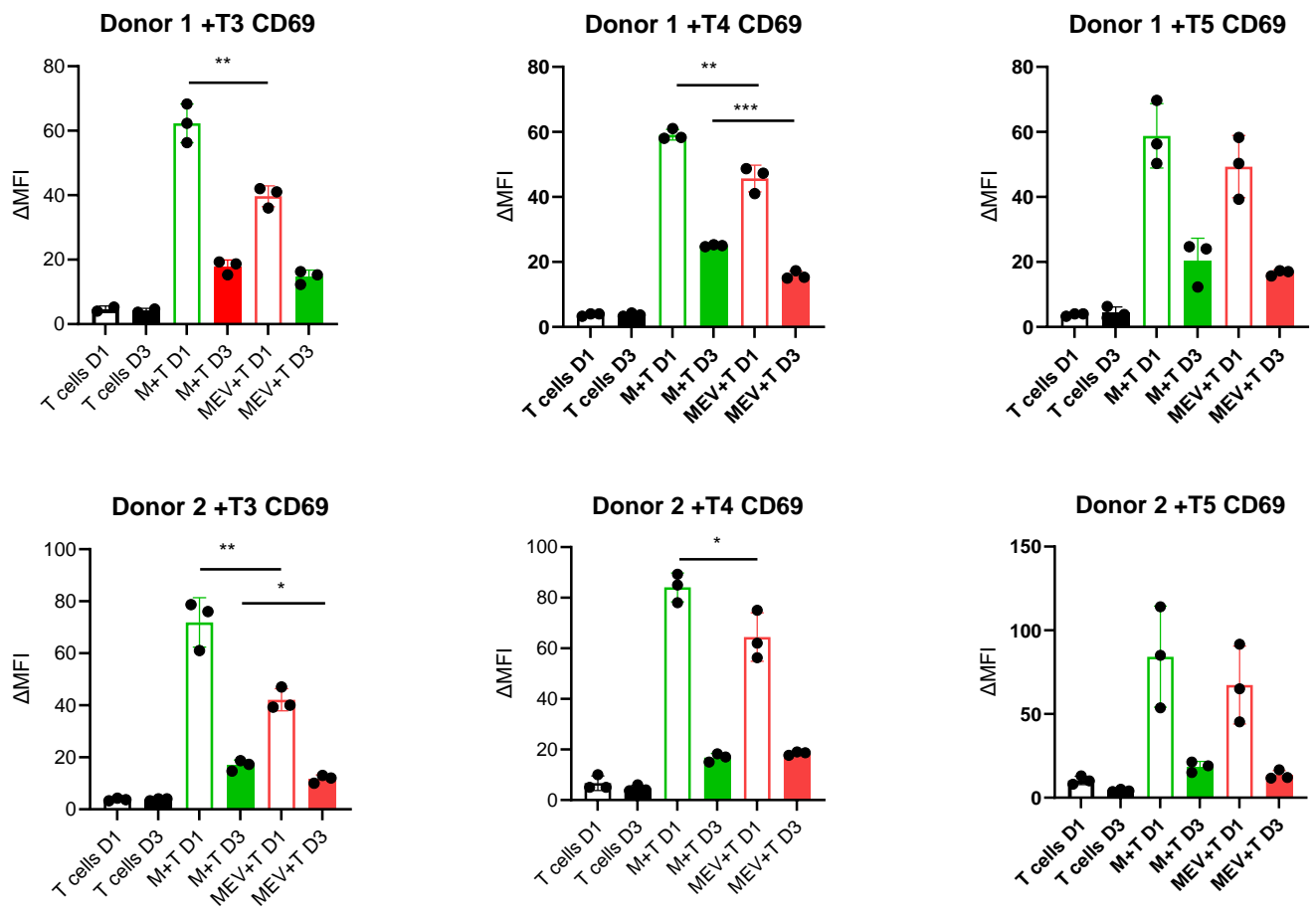**B**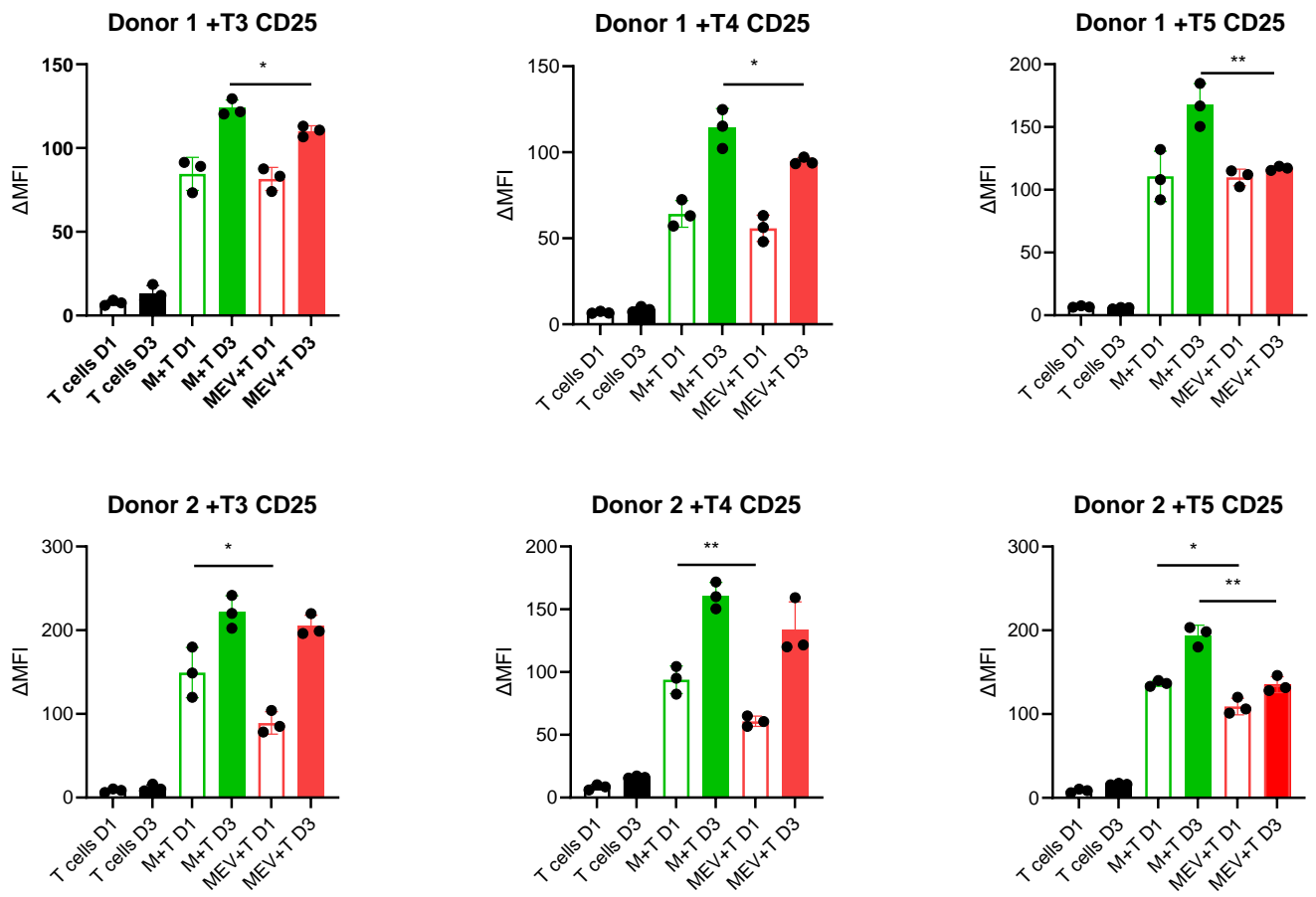

Supplement: Supplementary file 7 — Additional file 7: Supplementary Fig. 7. Human macrophages treated with hypoxic GBM-derived EVs have lower capacity to induce T cell activation markers after allogeneic stimulation. Human MDMs from two donors (Donor 1 and Donor 2) were cultured in the absence (M) or presence (MEV) of hypoxic GBM-derived EVs (1% EVs) for 7 days. On day 7 MDMs were cocultured with CD3+ T cells from three different donors (T3, T4 and T5) for 3 days. On day 1 and day 3 cells were harvested and the expression of (A) CD69 and (B) CD25 on T cells (CD4+ and CD8+) was measured by flow cytometry. Values are expressed as mean ± SD of three replicates from each MDM/T cell coculture, and comparisons were made using an unpaired t test. *p < 0.05, **p < 0.005, ***P < 0.001. [file 12964_2024_1523_MOESM7_ESM.pdf]

A

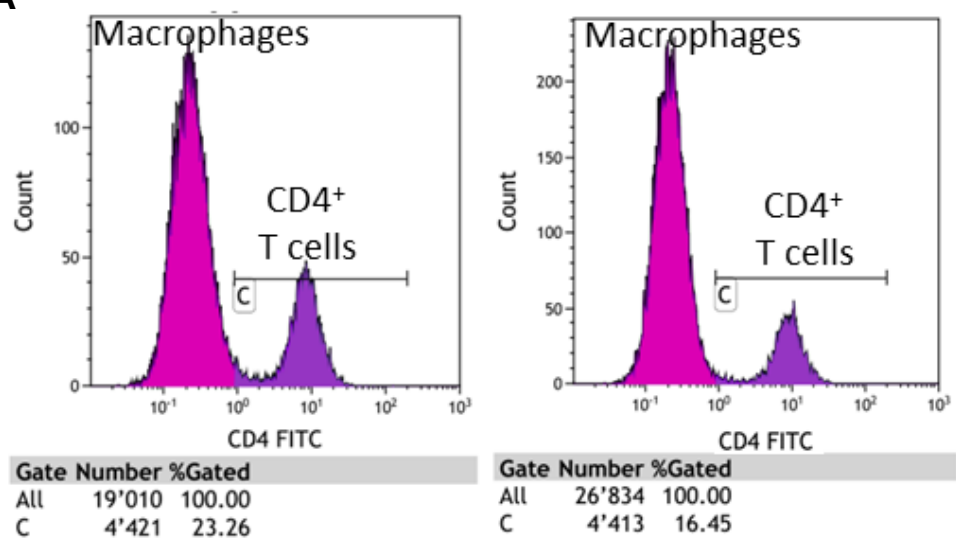

B

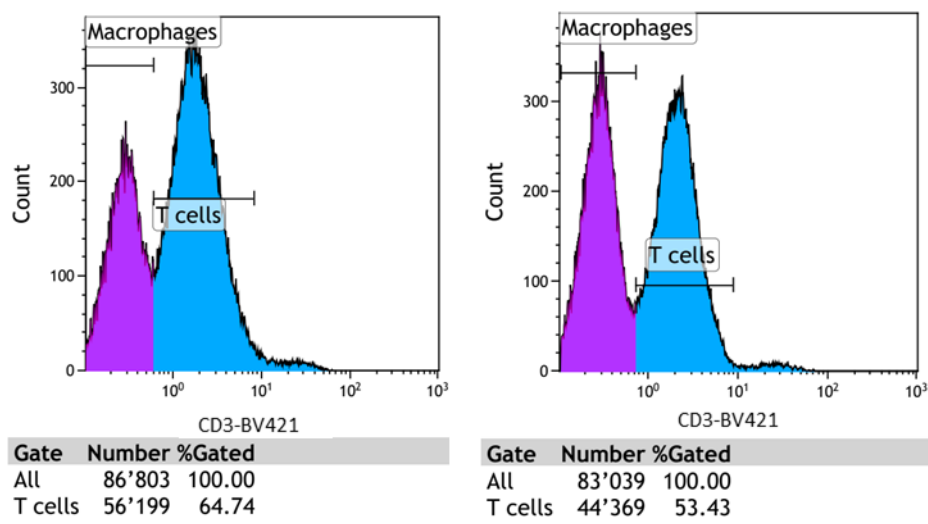

Supplement: Supplementary file 8 — Additional file 8: Supplementary Fig. 8. Representative flow cytometry readout for T cell migration assays shown in Fig. 7D, E. (A) Flow cytometry graph showing migration of murine CD4+ T cells from lower Transwell chamber in MO (control) and M0 + 1% EV conditions. (B) Flow cytometry graph showing migration of human CD3+ T cells from lower Transwell chamber in MO (control) and M0 + 1% EV conditions. [file 12964_2024_1523_MOESM8_ESM.pdf]
